# The two-pore channel TPC1 is required for efficient protein processing through early and recycling endosomes

**DOI:** 10.1038/s41598-017-10607-4

**Published:** 2017-08-30

**Authors:** Jan Castonguay, Joachim H. C. Orth, Thomas Müller, Faten Sleman, Christian Grimm, Christian Wahl-Schott, Martin Biel, Robert Theodor Mallmann, Wolfgang Bildl, Uwe Schulte, Norbert Klugbauer

**Affiliations:** 1 Institute of Experimental and Clinical Pharmacology and Toxicology, Faculty of Medicine, Albert-Ludwigs-University, Albertstrasse 25, 79104 Freiburg, Germany; 2 Institute of Physiology II, Faculty of Medicine, Albert-Ludwigs-University, Hermann-Herder-Strasse 7, 79104 Freiburg, Germany; 3 Logopharm GmbH, Schlossstrasse 14, 79232 March-Buchheim, Germany; 4 Center for Biological Signaling Studies (BIOSS), Schänzlestrasse 18, 79104 Freiburg, Germany; 5 Department of Pharmacy, Center for Drug Research and Center for Integrated Protein Science Munich (CIPSM), Ludwig-Maximilians-University, Munich, Germany

## Abstract

Two-pore channels (TPCs) are localized in endo-lysosomal compartments and assumed to play an important role for vesicular fusion and endosomal trafficking. Recently, it has been shown that both TPC1 and 2 were required for host cell entry and pathogenicity of *Ebola* viruses. Here, we investigate the cellular function of TPC1 using protein toxins as model substrates for distinct endosomal processing routes. Toxin uptake and activation through early endosomes but not processing through other compartments were reduced in TPC1 knockout cells. Detailed co-localization studies with subcellular markers confirmed predominant localization of TPC1 to early and recycling endosomes. Proteomic analysis of native TPC1 channels finally identified direct interaction with a distinct set of syntaxins involved in fusion of intracellular vesicles. Together, our results demonstrate a general role of TPC1 for uptake and processing of proteins in early and recycling endosomes, likely by providing high local Ca^2+^ concentrations required for SNARE-mediated vesicle fusion.

## Introduction

Two-pore channels (TPCs) are located in membranes of acidic intracellular organelles and share their basic domain architecture - each domain comprising six transmembrane segments - with TRP channels and voltage gated Na^+^ or Ca^2+^ channels. Functional TPC channels are assembled from two TPC protein subunits forming a pore that conducts mainly Ca^2+^ and Na^+^ (reviewed in refs 1–3). A hallmark of these channels is their specific activation by nanomolar concentrations of the second messenger NAADP^4^. In addition, recent work identified a specific phospholipid (Phosphatidylinositol 3,5-bisphosphate, PtdIns(3,5)P_2_) as potent activator of TPC channels^5^. Although the physiological functions of TPCs are not well understood, it is assumed that they are involved in the regulation of endocytosis and endo-lysosomal vesicle trafficking, i.e. of sorting and fusion events during protein uptake, protein recycling and degradation^6–8^. Trafficking between lysosomes, trans-Golgi network and the plasma membrane via endosomes and transport vesicles is tightly regulated by locally restricted Ca^2+^ release from acidic stores^2^. In addition to TPC1 and 2, other ion channels like TRPML1-3, TRPM2 and P2X4 channels have been shown to participate in endolysosomal Ca^2+^ release^7, 9–13^. The contribution of these distinct Ca^2+^ sources to specific intracellular trafficking processes and the molecular basis underlying their function, however, are presently not known.

We recently found that entry of filoviruses such as Ebola into host cells depends on TPCs and that genetic inactivation or pharmacological block of TPCs impairs virus replication and pathogenesis^14^. Ebola virions are internalized via macro-pinocytosis and follow a defined endosomal route to reach acidic compartments before viral genome release and replication. This intracellular processing is controlled by the activity of endolysosomal TPCs. Disruption of TPC1 or TPC2 either by gene knockout or by small interfering RNAs halted trafficking, trapped the virus particles in the endolysosomal network and prevented infection. Accordingly, the alkaloid tetrandrine blocking both TPC1 and TPC2 channels inhibited infection of macrophages, which represent the primary Ebola target cells and improved survival of infected mice^14^.

For a more systematic analysis of endosomal trafficking routes, bacterial protein toxins have been established as specific substrate tools^15^. These toxins are taken up by receptor-mediated endocytosis and hijack two different main endosomal routes to reach their final cytosolic destination. They either enter the cytosol from early and late endosomes (referred to as “short trip” toxins) or are transported via endosomal compartments to the Golgi apparatus and the ER in a retrograde manner (“long trip” toxins). The translocation of the first group of toxins from early and late endosomes into the cytosol is driven by ongoing acidification. The lethal factor (LF) of Anthrax toxin, Diphtheria toxin (DT) and *Pasteurella multocida* toxin (PMT) are examples for this uptake route. The second group of toxins including Cholera toxin (CT) is endocytosed, moved to late endosomes and transported in a retrograde manner along the secretory pathway to the ER^16, 17^. Successful passage of these toxins can be monitored by their modification of specific intracellular host cell target proteins. PMT permanently activates the Gα_q/11_, Gα_12/13_ and Gα_i_ family by deamidation of a specific glutamine residue in the α-subunit^18^. The deamidation can be monitored by immunoblot analysis utilizing a monoclonal antibody specifically detecting the deamidated form of the G protein^19^. CT activates heterotrimeric G proteins of the Gα_s_ family thereby stimulating the adenylyl cyclase leading in turn to elevated cAMP levels^20^. DT ADP-ribosylates the elongation factor-2 (EF-2) and thereby blocks protein translation leading to cell death^21^. And LF from anthrax toxin cleaves the N-terminus of MAPKK leading to diminished MAPK signaling^22^.

Whereas it had been demonstrated that TPC2 channels specifically localize to lysosomes and late-stage endosomes^2^, detailed studies on the distribution of TPC1 in endolysosomal (sub)compartments are lacking. For the latter, a series of GTPases of the Rab family and different classes of phosphoinositides (PIPs) have been described and widely used as immunocytochemical markers^23^. Both type of markers also act as platforms for the recruitment of effector proteins to membranes, which are crucial for key steps of intracellular trafficking processes. Their specificity for distinct endosomal compartments is, however, limited, as is the spatial resolution provided by confocal microscopy. To obtain a more detailed and unbiased view on the subcellular localisation of TPC1, image data analysis was done with two different methods, relative quantification of co-localisation and a Pearson correlation analysis (see Methods). In addition, we probed the molecular environment of native TPC1 channels in mouse kidney (where they are highly expressed) using an unbiased functional proteomic (AP-MS) approach. Identified interaction partners were verified in heterologous protein interaction assays and co-localization experiments.

We combined these approaches to uncover the cellular function and localization of TPC1 in endosomal compartments and to deduce its role in endosomal trafficking processes, also taking advantage of our TPC1 knockout mouse model^24^ as well as several independent human and murine TPC1 knockout cell lines which were generated by the CRISPR-Cas system^25^. We found that uptake and activation of protein toxins through the early endosomal route was impaired in TPC1 knockout cells or upon application of the TPC channel blocker tetrandrine, whereas toxins travelling to the endoplasmic reticulum were affected to a lesser extent. Consistently, TPC1 showed restricted localization to PtdIns(3)P, Rab4, Rab5 and Rab11-positive early and recycling endosomes. Functional proteomic analysis of native TPC1 channels in these organelles identified direct interaction with syntaxins 7, 8 and 12 suggesting a direct role of TPC1 as a Ca^2+^ source for SNARE-mediated vesicle fusion (and fission) in early and recycling endosomes.

## Results

To investigate the significance of TPC1 for endosomal trafficking we applied a set of bacterial toxins as route-specific model substrates to different wildtype and TPC1 knockout cells. TPC1 deficient cells were either derived from our knockout mice (mouse epidermal fibroblasts, MEFs)^24^ or generated from HeLa and J774 cells by using the CRISPR-Cas system^25^ as independent knockout models. Complete cellular knockout of TPC1 was verified by PCR amplification and sequence analysis of corresponding genomic DNA and Western blot analysis (Supplemental Figure S1). Cultures of these cells were incubated with either “short trip” or “long trip” toxins, and the effects of these toxins were compared between wild type and TPC1 knockout: MEFs were treated with cholera toxin (CT), or *Pasteurella multocida* toxin (PMT); HeLa cells were treated with CT or diphtheria toxin (DT). These toxin-cell combinations were necessary since toxin binding sites within the murine DT receptor differ from its human counterpart^26^; J774 macrophages were treated with Lethal Factor of anthrax toxin (LF) (see also Methods).

**Figure 1:**
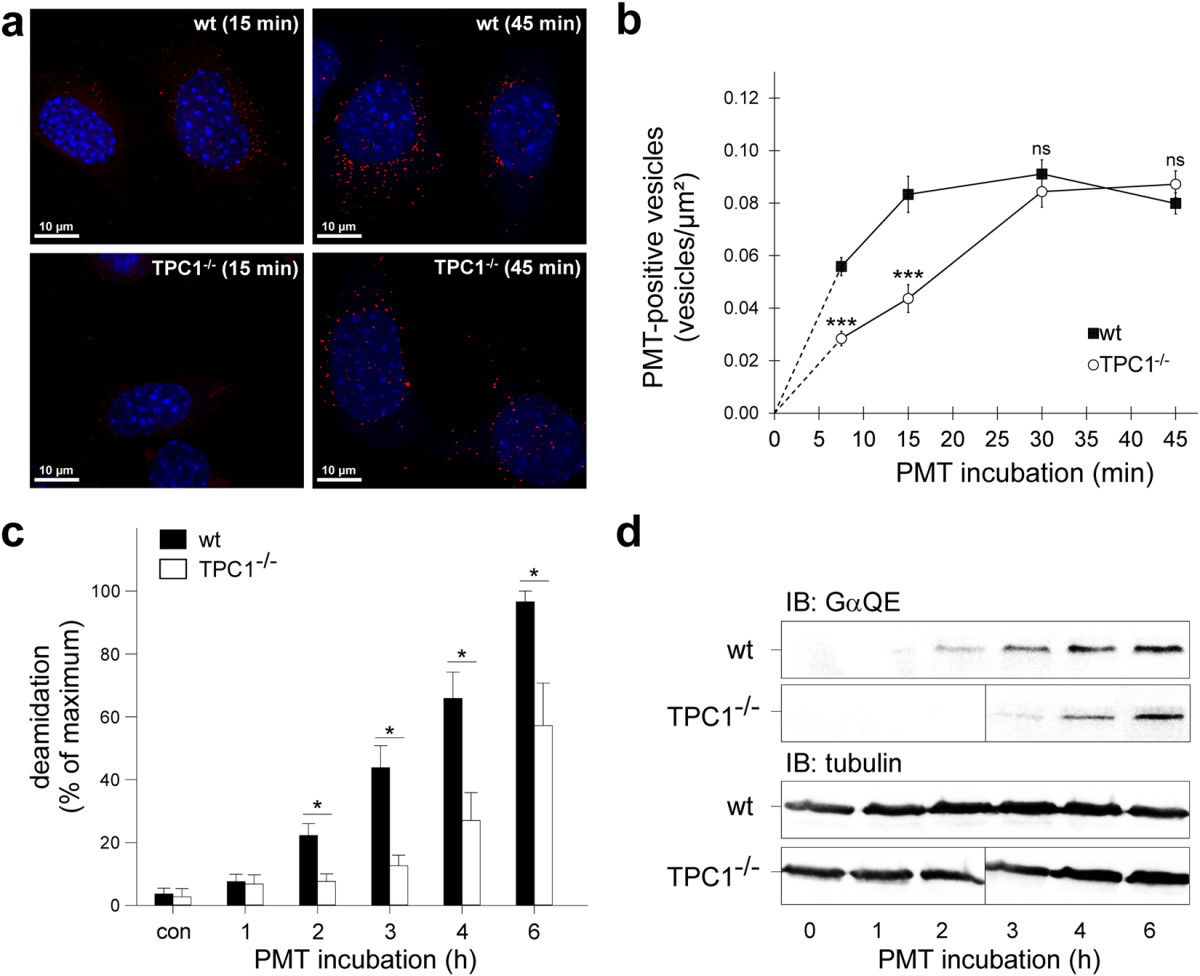
Deletion of TPC1 stalls uptake of *Pasteurella multocida* toxin. (**a**) WT-MEF or MEF deficient for TPC1 (TPC1^−/−^) were treated with Atto568-labeled toxin. Uptake was stopped via fixation after indicated times. Shown are representative pictures of MEF cells containing PMT-positive vesicles (red), nuclei stained with Hoechst (blue). (**b**) Time course of PMT uptake into MEF WT and MEF TPC1^−/−^ cells as in (**a**). For quantification of toxin uptake PMT positive vesicles were counted at indicated time points and related to cell size (n = 20). ***P < 0.001; ns *P* > 0.05 (Student t-test). (**c**,**d**) WT-MEF or MEF deficient for TPC1 were treated with PMT (100 pM) for indicated times and induced deamidation of heterotrimeric G proteins was analyzed by immunoblot (**d**) utilizing a deamidation specific Gα antibody (GαQE). Tubulin was used to verify equal loading. The results for TPC^−/−^ were combined from two gels using the same exposure time (dividing line). Degree of deamidation (**c**) was calculated from at least three independent experiments and depicted as percent of maximum deamidation in any given experiment (mean ± SEM) *P < 0.05; one-way analysis of variance (ANOVA) followed by Student-Newman-Keuls post hoc test.

**Figure 2:**
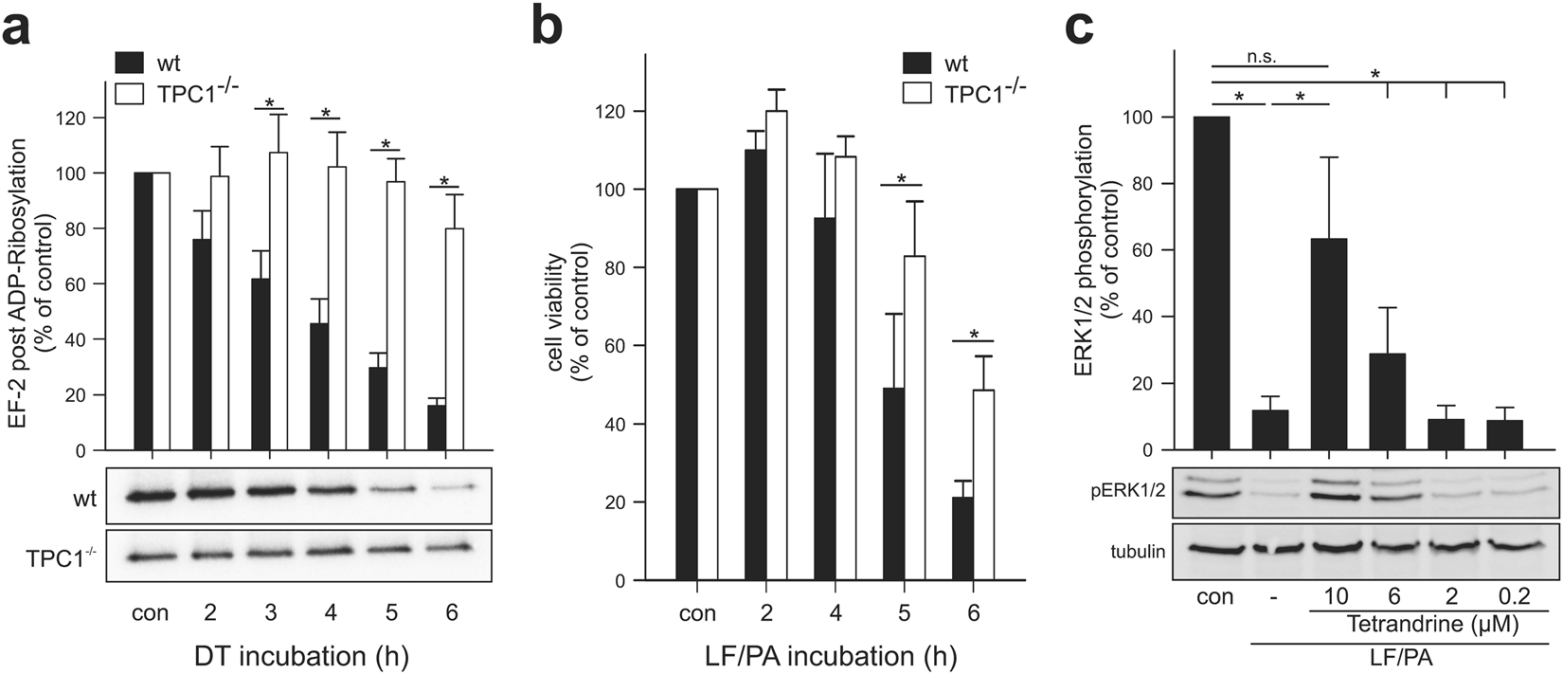
Activation of bacterial protein toxins is inhibited by deletion of TPC1. (**a**) WT-HeLa cells and TPC1-knock-out HeLa cells (TPC1^−/−^) were incubated with DT (100 pM) for indicated times. Thereafter post-ADP-ribosylation of EF-2 utilizing ^32^P-NAD^+^ was performed. At least three independent experiments were performed (mean ± SEM). (**b**) Lethal toxin-induced cell death in WT-J774 macrophages and TPC1-deficient J774 macrophages. Cells were incubated with the binary lethal toxin consisting of protective antigen (PA, 0.5 µg/ml) and lethal factor (LF, 0.3 µg/ml) for indicated times. Thereafter, cell viability was determined utilizing the cell titer blue assay and calculated in percent of untreated (con) cells (n ≥ 3, mean ± SEM). (**c**) Tetrandrine inhibits lethal toxin-induced MAPK cleavage in J774 macrophages. Cells were pre-treated with indicated concentrations of tetrandrine for 30 min. Thereafter J774 macrophages were incubated for 3 h with the binary lethal toxin consisting of protective antigen (PA, 0.5 µg/ml) and lethal factor (LF, 0.3 µg/ml). Cell lysates were prepared and activation of ERK1/2 was determined by Western blot using phospho-specific ERK1/2 antibody (mAb #4370, Cell Signaling). Equal loading was verified by detection of tubulin. Quantification (upper panel) was calculated from at least three independent experiments and depicted as percent of maximum ERK1/2 phosphorylation in any given experiment (mean ± SEM); *P < 0.05; n.s. *P* > 0.05; one-way analysis of variance (ANOVA) followed by Student-Newman-Keuls post hoc test.

We first investigated cellular toxin uptake using fluorescently (Atto585)-labelled PMT, and the number of PMT-positive vesicles per cell size (area in square µm) was counted at different time points. As shown in Fig. 1a PMT uptake by MEFs was efficient reaching a maximum within 30 min. This uptake was significantly slower in MEF TPC1^−/−^ cells (Fig. 1b). We also used the TPC channel blocker tetrandrine and found a concentration dependent inhibition of toxin uptake (Supplemental Figure S2). Furthermore, incubation with PMT led to a time dependent deamidation of heterotrimeric G proteins first observable after 2 h. In TPC1 deficient MEFs deamidation was detectable about 2 h later and reached only about 50% as compared to wild type MEFs (Fig. 1c,d). Likewise, HeLa wild type and TPC1 knockout cells were treated with DT, and the toxin activity was measured using an EF-2 post-ADP-ribosylation assay. Cells were intoxicated with DT for indicated times followed by lysate preparation. Hereafter an *in vitro* post ADP-ribosylation with newly added DT and ^32^P-labelled NAD^+^ was performed to detect the unmodified EF-2 fraction (see also Methods). As shown in Fig. 2a, EF-2 was modified almost completely within 6 h upon toxin incubation in wildtype cells, whereas almost no EF-2 ribosylation could be measured in the respective TPC1 knockout during this time span (Fig. 2a).

Next, we studied anthrax toxin from *Bacillus anthracis* that attacks the immune defense of the host by targeting immune cells^27^ in J774 wildtype and TPC1 deficient macrophages (Fig. 2b). The cytotoxic effect of LF/PA was monitored over a period of 6 h in which 80% of wildtype macrophages died. In TPC1-deficient J774 macrophages the toxic effect of LF/PA was delayed. To exclude target-unrelated effects in the TPC1 knockout, we used the TPC channel blocker tetrandrine^14^ as a knockout mimic in wildtype macrophages. This inhibitor decreased the effect of LF/PA in a dose-dependent manner (half-maximal effect between 6 and 10 µM) as determined by the phosphorylation status of the MAPK ERK1/2 (Fig. 2c).

The “long-trip” surrogate CT stimulated cAMP accumulation measured as a reduced ATP-dependent luciferase activity within 3 h of treatment (Supplemental Figure S3a,b). In contrast to the three tested “short-trip” toxins, CT activity in both MEFs and HeLa cells showed very little quantitative difference between wildtype and the respective TPC1 knockout cells. A previous study observed a reduction of CT accumulation in the Golgi apparatus in TPC1 knockout cells after a chase time of two hours^28^. However, it remains open if this relates to an altered biological activity. In summary, deletion of TPC1 reduced or attenuated the uptake and/or biochemical activity of all short-trip toxins entering via early or recycling endosomes, whereas the effect of toxins undergoing retrograde transport from late endosomes to the ER remained largely unchanged.

**Figure 3:**
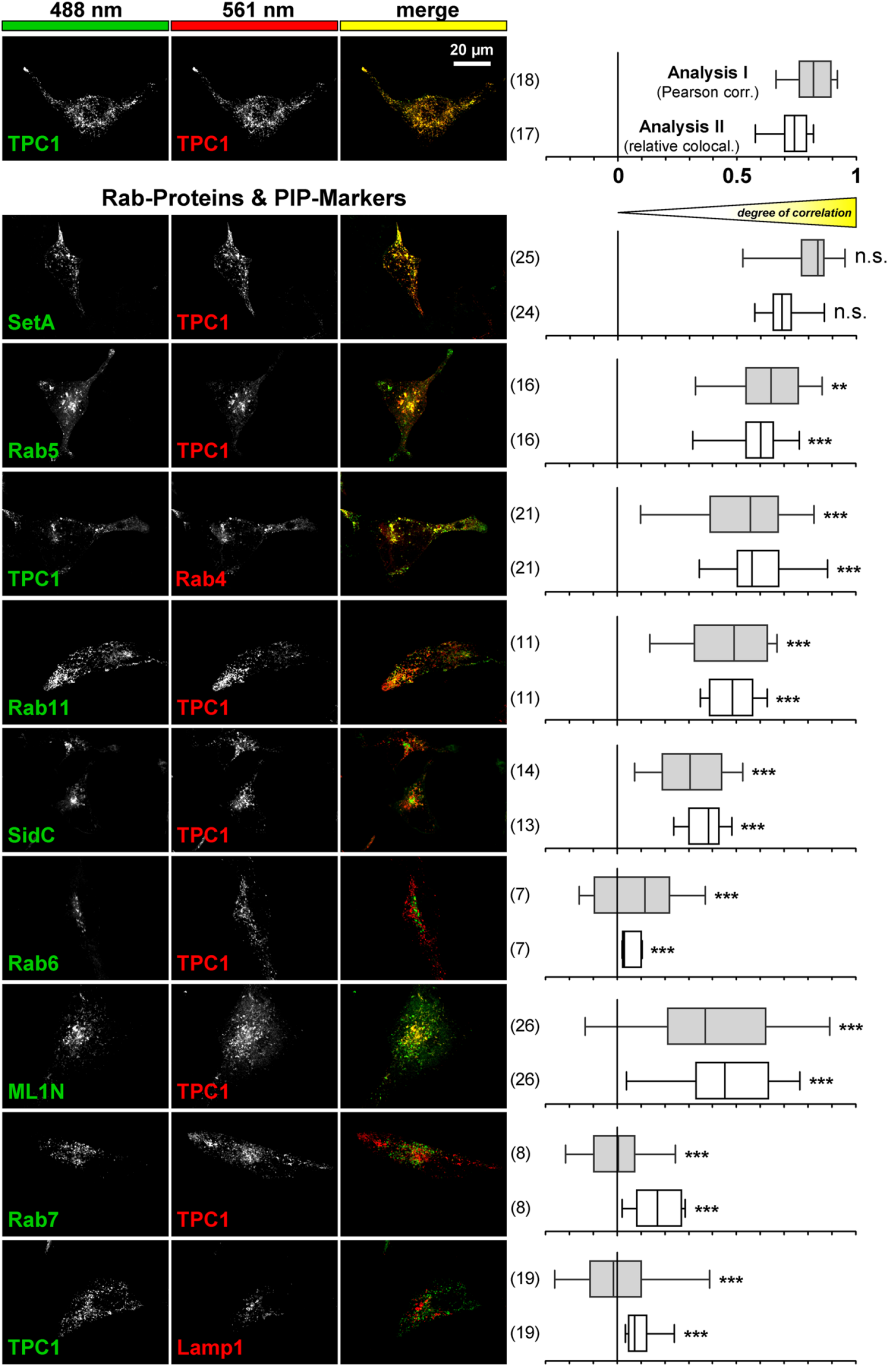
Co-localization and correlation analysis of TPC1 with a set of nine intracellular markers in HeLa cells. The first and second column show the fluorescence signal at 488 and 561 nm and indicate the used constructs or staining, respectively. The merge of the green and red fluorescence channel is demonstrated in the third column. The last column summarizes and quantifies the results either as co-localization (white bars) or as Pearson correlation (grey bars) (Methods), n = number of cells investigated. As a reference we used co-expression of TPC1-EGFP and TPC1-tomato. ***P < 0.001; **P < 0.01; *P < 0.05; n.s. *P* > 0,05.

We further investigated the localization of TPC1 in the compartments of the secretory pathway by immunocytochemistry. We used a set of 10 different established markers for distinct populations of intracellular vesicles either co-transfected or specifically stained in HeLa cells expressing fluorescently tagged TPC1 (Fig. 3). To increase resolution and specificity of this approach, distribution of fluorescence was read out by confocal microscopy and evaluated quantitatively across multiple cells using Pearson correlation of fluorescence intensity profiles along cell sections. In order to obtain a most detailed view on the subcellular localization of TPC1 we performed a second analysis, taking into account potentially different expression levels of the used markers (see methods). Rab-GTPases are membrane-associated small GTPases and belong to the most widely used markers to define intracellular compartments^23^. As shown in Fig. 3, Rab4, Rab5 and Rab11 showed the strongest correlation with TPC1 distribution with correlation/co-localization values (Pearson correlations and relative co-localization values: Rab4: 0.54 ± 0.18 and 0.59 ± 0.12; Rab5: 0.64 ± 0.16 and 0.59 ± 0.10; Rab11: 0.46 ± 0.19 and 0.48 ± 0.09; number of cells are each indicated in Fig. 3) close to those measured by TPC1-GFP/TPC1-tomato as a positive reference (0.82 ± 0.08 and 0.74 ± 0.07). Rab5 labels early endosomes and Rab11 marks recycling endosomes, whereas Rab4 may represent a marker of transitional vesicles (reviewed in ref. 29). In contrast, Rab7 which is present in late endosomes and multivesicular bodies, but also in autophagosomes and lysosomes, the trans-Golgi marker Rab6 and autophagosome marker LC3 as well as the lysosomal marker Lamp1 did neither co-localize nor correlate with TPC1 (Pearson correlations and relative co-localization values: Rab7: 0.00 ± 0.14 and 0.17 ± 0.1; Rab6: 0.10 ± 0.19 and 0.05 ± 0.04; LC3: 0.24 ± 0.18 and 0.17 ± 0.11; Lamp1: 0.01 ± 0.16 and 0.10 ± 0.06).

Besides investigating Rab-GTPases, we extended colocalization experiments with labelling of selected phosphoinositide phosphates (PtdInsPs or, briefly, PIPs). Some PIP species are found enriched in distinct vesicle populations where they have been shown to be functionally important, i.e. for effector recruitment and as determinants for trafficking and vesicle fusion specificity, much alike and in concert with Rabs^23^. PtdIns(3)P is enriched in early endosomes^30^ and also present in autophagosomes, PtdIns(4)P shows a broad localization ranging from Golgi and trans-Golgi network to secretory vesicles and finally PtdIns(3,5)P_2_ is predominantly found in late endosomes, multivesicular bodies and lysosomes^23^. For specific detection of these different PIP species we used specific bacterial PIP binding proteins that have been established as superior tools for classification of subcellular organelles^31, 32^. We took advantage of fluorescently labelled *Legionella pneumophila* proteins SetA and SidC that specifically bind to PtdIns(3)P and PtdIns(4)P, respectively. Indeed, SetA demonstrated the strongest co-localization and correlation of all markers used in our study. Quantification revealed no significant difference compared to our internal TPC1/TPC1 reference (SetA: 0.81 ± 0.11 and 0.69 ± 0.07). In contrast, SidC only showed a very low co-localization and correlation with TPC1 (SidC: 0.31 ± 0.14 and 0.36 ± 0.08).

Noteworthy, these experiments also revealed marked difference in the broadness of marker distributions pointing to different degrees of specificity and/or spatial extension of the labelled compartments. Accordingly, our data suggest a restricted, well-defined localization of TPC1 in early endosomes and potentially recycling endosomal vesicles, but not in the Golgi, trans-Golgi network or secretory vesicles. Furthermore, no overlap was observed with lysosomal compartments that have been shown to contain TPC2^33^.

PtdIns(3,5)P_2_ has been reported to activate both TPC subtypes either directly^5^ or indirectly^34^. We therefore employed a PtdIns(3,5)P_2_ probe consisting of the cytosolic N-terminal tandem repeats of TRPML1 (Mucolipin 1) fused to an EGFP-tag^35^. In line with previous findings^35^, we observed broad and heterogenous distribution patterns with additional non-vesicular or cytosolic localization. Whereas some cells exhibited a broad and more ramified pattern of PtdIns(3,5)P_2_ with a high degree of co-localization and correlation with TPC1, other cells showed a more punctate PtdIns(3,5)P_2_ staining with only very low co-localization and correlation with TPC1 (Supplemental Figure S4; combined data shown in Fig. 3). Thus, PtdIns(3,5)P_2_ may neither be a specific nor a reliable marker for localization of TPC1.

**Figure 4:**
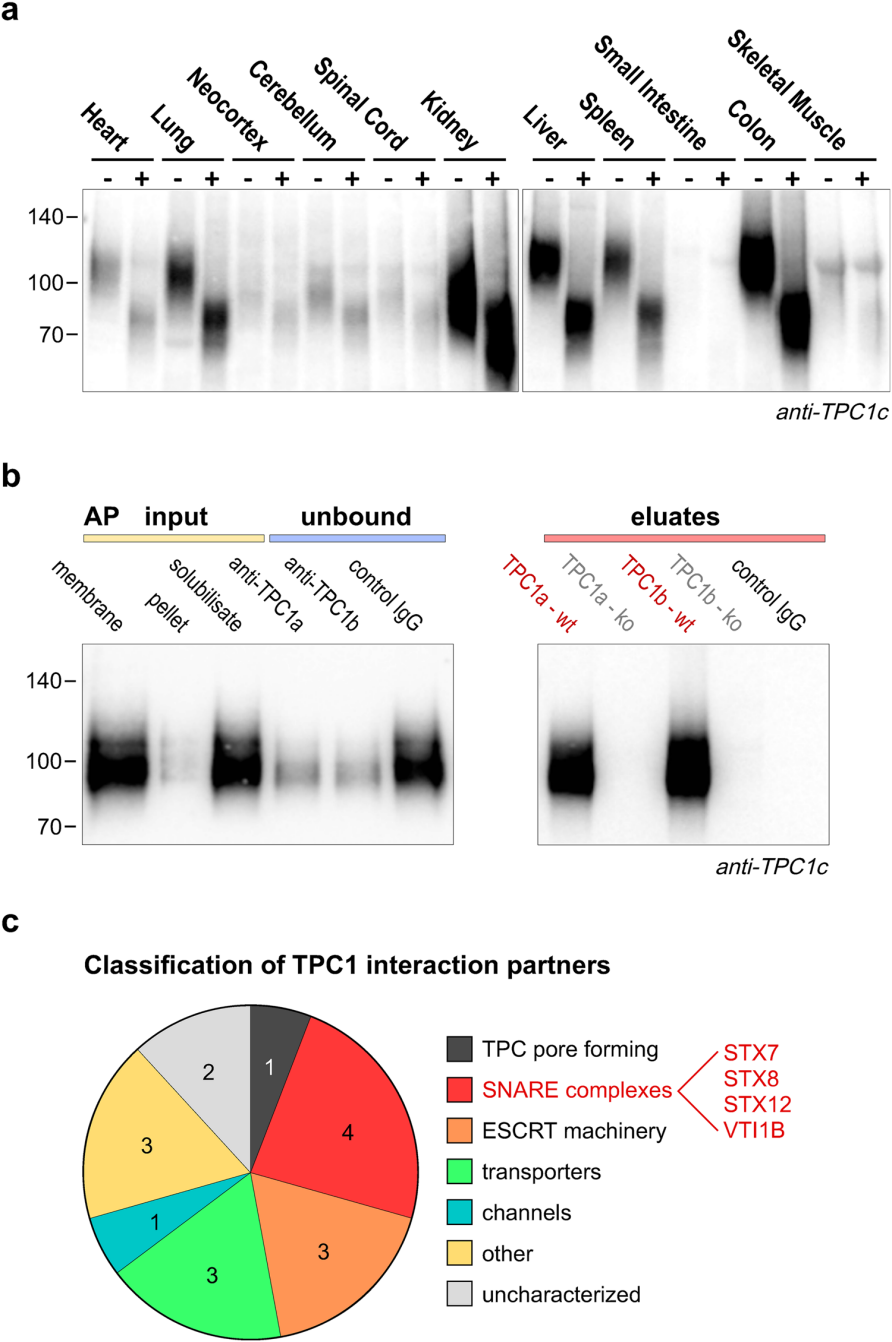
Native source-based AP-MS analysis of TPC1. (**a**) Western blot assay probing total membranes isolated from the indicated mouse tissues for TPC1 expression (representative example of three replicate experiments; tissues were separated on two gels using the same exposure time). Each lane resolved 3 µg of protein either with ( + ) or without (−) pretreatment with PNGase F to remove N-glycosylation. Note the tissue-dependent heterogeneity in glycosylated TPC1 species. (**b**) Antibody-based affinity purification (AP) of TPC1 from mouse kidney endosome-enriched membrane fractions solubilized with CL-72. Western blots were stained with anti-TPC1 (#3526; 0.1 µg/ml final concentration)/HRP-conjugated anti-rabbit and developed with ECL + . Left: Lanes 1 to 6 show (1) solubilisate before centrifugation, (2) pellet of insolubilized material, (3) solubilisate after centrifugation, (4) solubilisate after CoIP using antiTPC1a (#832), (5) solubilisate after CoIP using antiTPC1b (#836) and (6) solubilisate after CoIP using control IgG. Right: Lanes 1 to 4 show CoIP eluates from wild type and TPC1 knockout using indicated antibodies. (**c**) Proteins specifically and consistently co-purified with both anti-TPC1 antibodies, functionally grouped according to their assignment in the UniprotKB/Swiss-Prot database (see also Supplemental Table S1).

We finally aimed to study the molecular environment of native TPC1 channels in endosomal compartments using an AP-MS approach^36^. The latter was enabled by the development of TPC1-specific antibodies^24^ and the established TPC1 knockout mouse model^24^ serving as a stringent co-purification specificity control. To select a suitable tissue source for this low-abundant target, we first used a Western blot screen of membranes prepared from prominent/main organs isolated from mouse. As shown in Fig. 4a, TPC1 is broadly expressed with higher density in epithelial tissues like lung, colon and, most prominently, kidney. We then established a protocol for isolating membrane fractions enriched for TPC1 from kidney using sucrose gradient ultracentrifugation. Combination of two consecutive centrifugation steps of a precleared tissue lysate on a discontinuous density gradient (further details in Materials and Methods) allowed for isolation of a single fraction containing an enriched pool of TPC1-positive vesicles (Supplemental Figure S5a), consistent with its restricted localization to specific endosomal compartments. This vesicle fraction was solubilized under mild conditions (ComplexioLyte 72, ref. 37) and subjected to two antibody-based affinity purifications. Efficient affinity-capture of solubilized TPC1 in APs was verified by Western blot analysis (Fig. 4b). The major part of the AP eluates was subjected to high-resolution LC-MS/MS analysis providing information on both quantity and identity of peptides. These analyses identified significant amounts of TPC1 with either antibody-AP (relative sequence coverage around 50%; Supplemental Figure S5b,c). In contrast, no peptides specific for TPC2 were identified strongly arguing against the formation of TPC heteromers in native endosomal membranes.

Using control anti-TPC1 APs from corresponding TPC1 knockout membranes, we identified a set of 16 proteins as consistently and specifically co-purified partners of TPC1 (Fig. 4c and Supplemental Table S5) from a total pool of >800 identified and quantified proteins (Supplemental Figure S5b). These putative TPC1 interaction partners, mainly integral membrane proteins, can be grouped into several classes based on functional assignment in the UniProtKB/Swiss-Prot database. These comprised a number of transporters, channels, and various other functions including a number of essentially uncharacterized proteins (Fig. 4c and Supplemental Table S1). Strikingly, a major portion of these TPC1 partners has been implicated in vesicle fusion and trafficking processes, either as part of SNARE complexes or the ESCRT machinery. Since TPC1 channels have been implicated as Ca^2+^ sources triggering vesicle fusion events, we further tested whether they directly interact with SNARE proteins.

**Figure 5:**
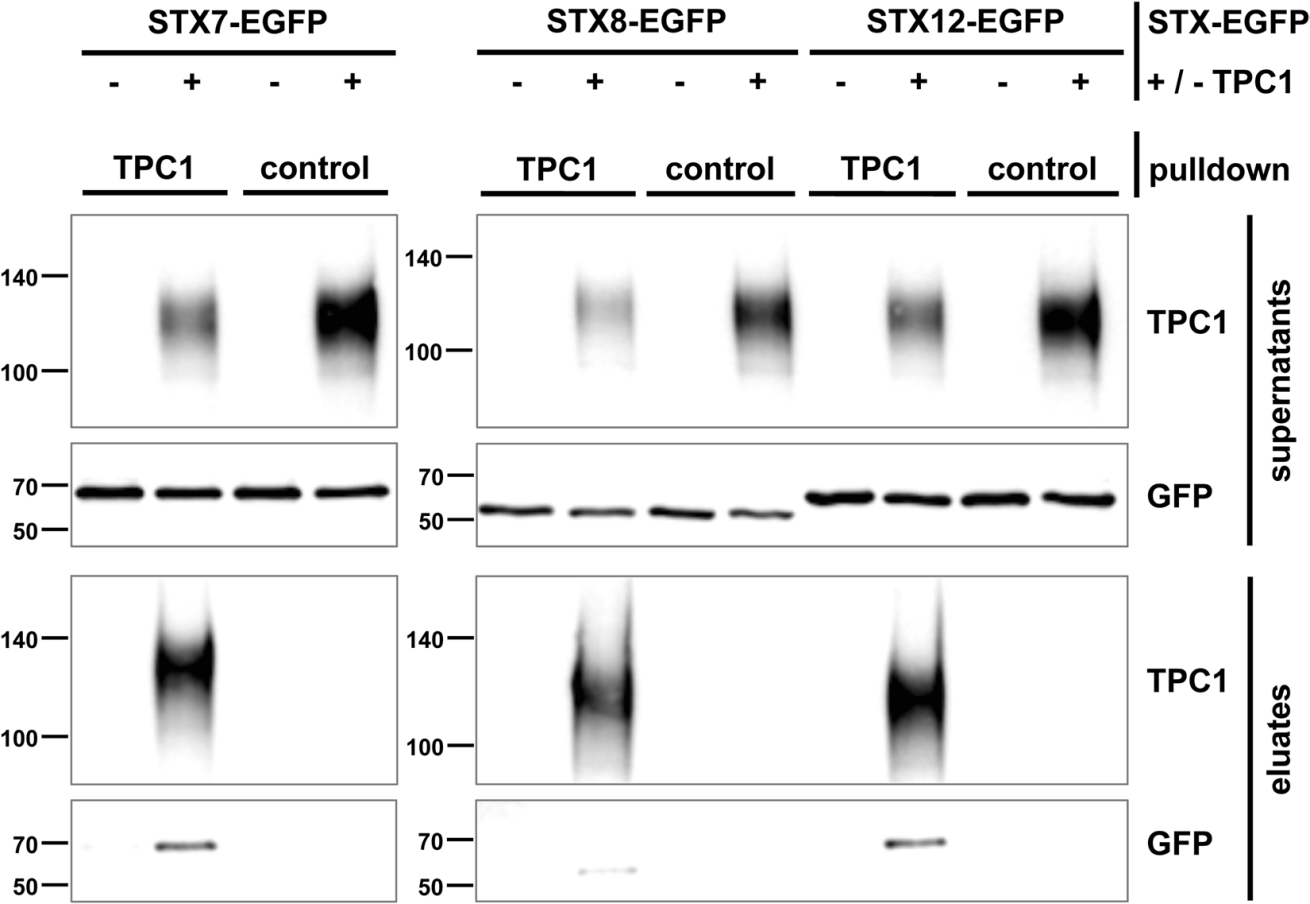
Co-immunoprecipitation of TPC1 with EGFP-tagged syntaxins STX-7, STX-8 and STX-12 heterologously expressed in MDCK cells. Pulldowns were performed with either anti-TPC1 or control IgG antibody as indicated. The two upper panels show the supernatants (unbound fraction), the lower lines the eluates using either anti-TPC1 or anti-GFP antibodies (Abcam #6658). Separate gels are framed.

**Figure 6:**
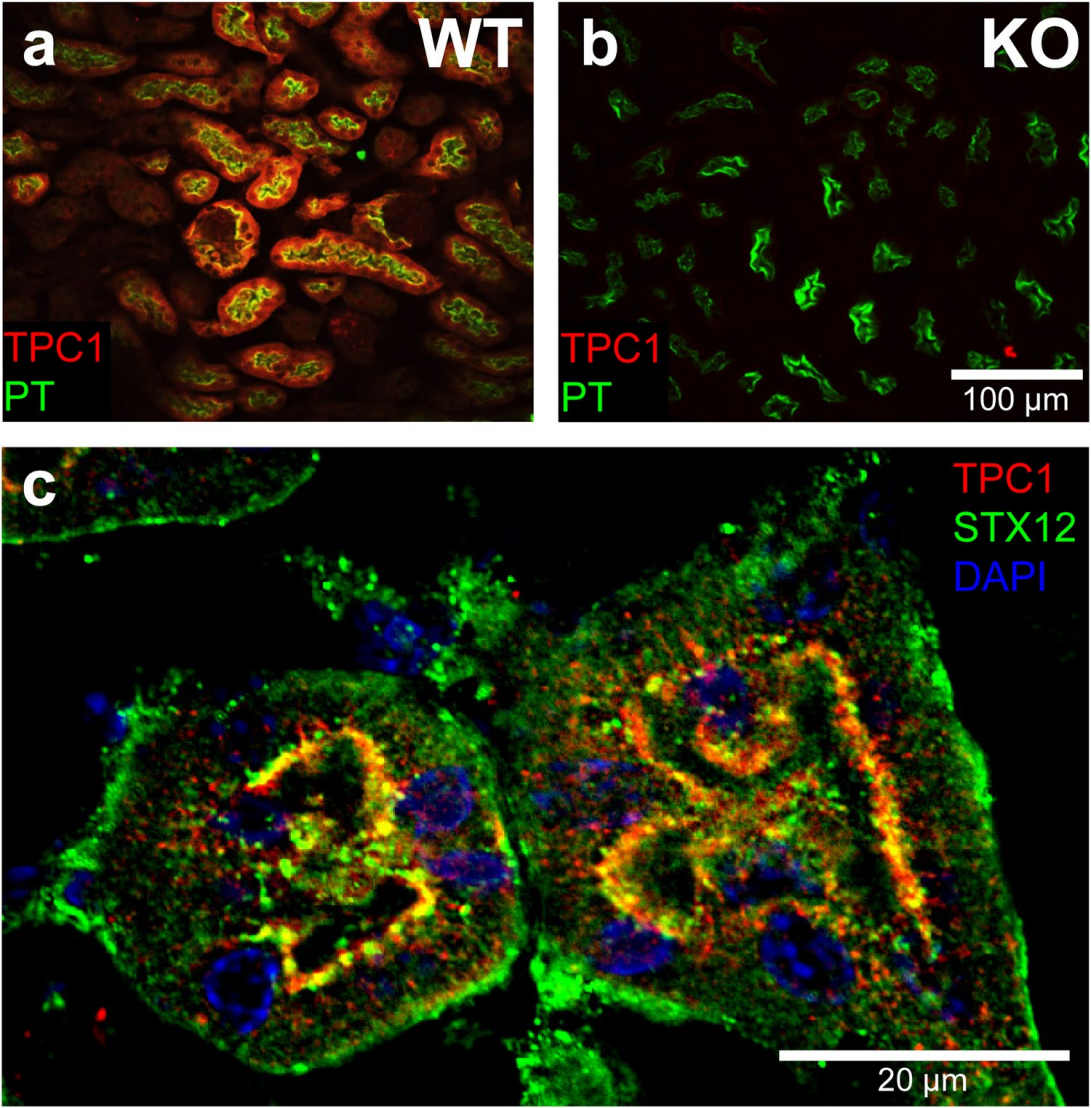
Immunohistochemical detection of TPC1 in kidney and co-localization with syntaxin-12. (**a**) TPC1 (red) is predominantly expressed in the proximal tubule as indicated by co-staining with the marker *Lotus tetragonolobus* lectin (green) (Vector laboratories #B1325). (**b**) Control staining using a kidney section from a TPC1 knock-out mouse. Staining with antibodies as in (**a**), scale bar = 100 µm for (**a**) and (**b**). (**c**) Co-localization of TPC1 (red, anti-TPC1, generated by Gramsch Laboratories) with syntaxin-12 (green, anti-STX12 Synaptic Systems) at the apical membrane of kidney proximal tubules. DAPI staining in blue; scale bar = 20 µm.

Subsequent to heterologous co-expression of TPC1 with different types of syntaxins, we performed co-immunoprecipitation studies with immobilized anti-TPC1 antibody. We found that the Qa-SNAREs syntaxin-12 and syntaxin-7 directly bound to TPC1 (Fig. 5). Additionally, we observed weak co-precipitation of the Qc-SNARE protein syntaxin-8. In contrast, we could not specifically co-immunoprecipitate the Qc-SNARE syntaxin-6 which is typically found in syntaxin-12 containing SNARE complexes. To confirm our findings in native tissue, we performed co-localization experiments of TPC1 and syntaxin-12 using tissue sections from mouse kidney. First, we investigated the expression pattern of TPC1 in kidney tissue by immunohistochemistry using kidneys from TPC1 knockout mice as controls (Fig. 6a,b). Co-staining with *Lotus tetragonolobus* lectin identified proximal tubule cells with the highest expression level of TPC1 (Fig. 6a)^38, 39^. Then, cortical sections from kidneys were co-stained with anti-TPC1 and anti-syntaxin-12 antibodies. As shown in Fig. 6c, TPC1 and syntaxin-12 were found strongly co-localized at the apical membrane of proximal tubular cells. In addition, syntaxin-12 was also detected in TPC1-negative vesicles and along the basolateral membranes.

Taken together, we conclude that TPC1 directly interacts and co-localizes with a subpopulation of syntaxin-12 and may fuel early endosomal membrane fusion processes mediated by this SNARE protein.

## Discussion

In this study we combined three experimental approaches to investigate the precise organellar localization and cellular function of TPC1 channels. First, using a panel of bacterial toxins as established substrates for distinct endosomal trafficking routes, we found that knockout of TPC1 specifically affected toxin entry through early and late endosomes in line with results obtained previously for the entry of Ebola viruses. Second, detailed co-localization analysis of fluorescence-tagged TPC1 and different subcellular markers confirmed that TPC1 is predominantly localized to early, recycling – and to a lesser extent also late - endosomal compartments. And third, preparation of the latter from kidney was used as source material for antibody-based affinity purification of native TPC1 channels followed by LC-MS/MS analysis identifying 16 putative TPC1 interactions partners. Among these, SNARE proteins syntaxin 7, 8 and 12 were confirmed as direct interactors of TPC1 providing a possible molecular explanation for how local Ca^2+^ signals elicited by TPC1 might efficiently trigger fusion of endosomal vesicles.

These fusion events direct the processing and trafficking of endocytosed material in endolysosomal compartments and therefore play a critical role for regulating protein turnover, phagocytosis but also for entry and activation of certain viruses and bacterial toxins. Knockout of TPC1 consequently was found to partly inhibit or delay uptake (Fig. 1a,b) and activation (Figs 1c and 2) of several bacterial toxins. These partial effects could be explained by alternative Ca^2+^ sources in these compartments enabling vesicle fusion such as TRPML or P2X4 channels^7, 9, 11, 12, 40^, or fusion events independent from local Ca^2+^ signals. In any case, a small residual rate of endosomal fusion events might still be sufficient for the measured activity of these highly effective toxins.

A precise description and classification of TPC1 containing compartments is central to the understanding of the intracellular role of TPC1. Since TPC1 channels are expressed at rather low densities, we analyzed subcellular localization of heterologously expressed tagged TPC1. In contrast to previous studies, however, we used a panel of ten established markers and provided quantitative analyses of co-localization and correlation utilizing co-expression of TPC1-EGFP and TPC1-tomato for reference. Due to the individual limitations of some of the markers used in the literature, we are convinced that only the combined dataset gives a reliable view of the intracellular expression of TPC1. According to our results, TPC1 can be attributed to early endosomes (indicated by Rab4, Rab5 and the PI(3)P-marker) and recycling endosomes (indicated by Rab11 and Rab4), but may be also expressed in low amounts in other compartments. Our results are in line with previous studies from Ruas and colleagues who identified the long isoform TPC1A in the same compartments, but found a shorter transcript TPC1B in late endosomes^28^. This may suggest that the transition from early to late endosomes might be accompanied by a shift from TPC1A via TPC1B to TPC2 positive compartments. More specific and sensitive antibodies against subcellular markers may allow for detailed analysis of TPC1 localization also in native cell types that express higher levels of TPC1 such as renal proximal tubular cells (Fig. 6).

Within the group of subcellular markers, we also analyzed co-localization of PI(3,5)P_2_ with TPC1. This phospholipid is of particular interest since it is considered to be either a direct activator or a permissive factor for TPC activation. Moreover, PI(3,5)P_2_ has also been reported to play a role for activation of TRPMLs^9, 41, 42^ and ryanodine receptors^43^ and even might act as a regulator of TORC1^44, 45^. PI(3,5)P_2_ localization is highest in late endosomes and lysosomes arguing against a specific functional role for TPC1 in early endosomes and recycling endosomes. Our observation pointed to a rather bimodal nature of its localization. On one side, cells showed a low co-localization of TPC1 with the PI(3,5)P_2_ probe which appeared in a more pronounced and clearly punctuated manner, in line with published work. Opposed to that, a subpopulation of cells showed a rather broad, more ramified and less pronounced PI(3,5)P_2_ probe localization, which displayed a high degree of co-localization with TPC1. We speculate, that these distributions might be explained by the differential synthesis and/or localization of PI(3,5)P_2_.

In the next step, we carefully optimized our AP-MS approach with regard to the generally low expression density of TPC1 in native tissues. Screening tissues with a knockout-validated specific anti-TPC1 antibody revealed highest expression of TPC1 in fluid resorbing epithelial tissues such as kidney and colon. In all tissues investigated, TPC1 showed excessive and heterogeneous N-glycosylation (Fig. 4a) which was previously described also for recombinantly expressed TPC1^4, 46^. Kidneys from mouse were fractionated by sucrose density gradient centrifugation to obtain membranes highly enriched in TPC1 (Supplemental Figure S4a). Affinity purification from these membranes under a mild solubilisation condition was highly effective (>90%, Fig. 4b) but still provided a limited signal-to noise with TPC1 protein abundance remaining within the range of multiple background proteins as quantified by mass spectrometry (Supplemental Figure S5b). Nevertheless, LC-MS/MS analysis identified a number of TPC1 peptides (Supplemental Figure S5c and Table S1) and - using control measurements of APs from TPC1 knockout membranes – 16 TPC1 candidate interaction partners (Supplemental Table S1). These results represent the first, although not comprehensive, proteomic analysis of native TPC1 channels (with only a minor overlap with proteomic results obtained recently from a recombinant approach^47^) and provided several striking findings: First, no peptides specific for TPC2 were identified, contrasting the results from heterologous co-expression experiments suggesting TPC1/2 heteromer formation^48^. Second, the vast majority of the candidate interaction partners had been assigned endo-lysosomal function and/or localization in the UniProtKBt/Swiss-Prot database (Supplemental Table S1) in line with the results from functional and co-localization experiments. And third, syntaxins 7, 8 and 12 from the prominent group of SNARE proteins were identified as direct binding partners of native TPC1 pointing to formation of Ca^2+^ nano-domains of TPC1 with the vesicle fusion apparatus. In this context it has been reported that early endosomal fusion processes rely on SNARE complexes containing syntaxin-12, whereas SNARE complexes mediating late endolysosomal fusions include syntaxin-7 and syntaxin-8^49–51^. Further detailed experiments are needed to elucidate the role of TPC1 in these fusion events.

**Figure 7:**
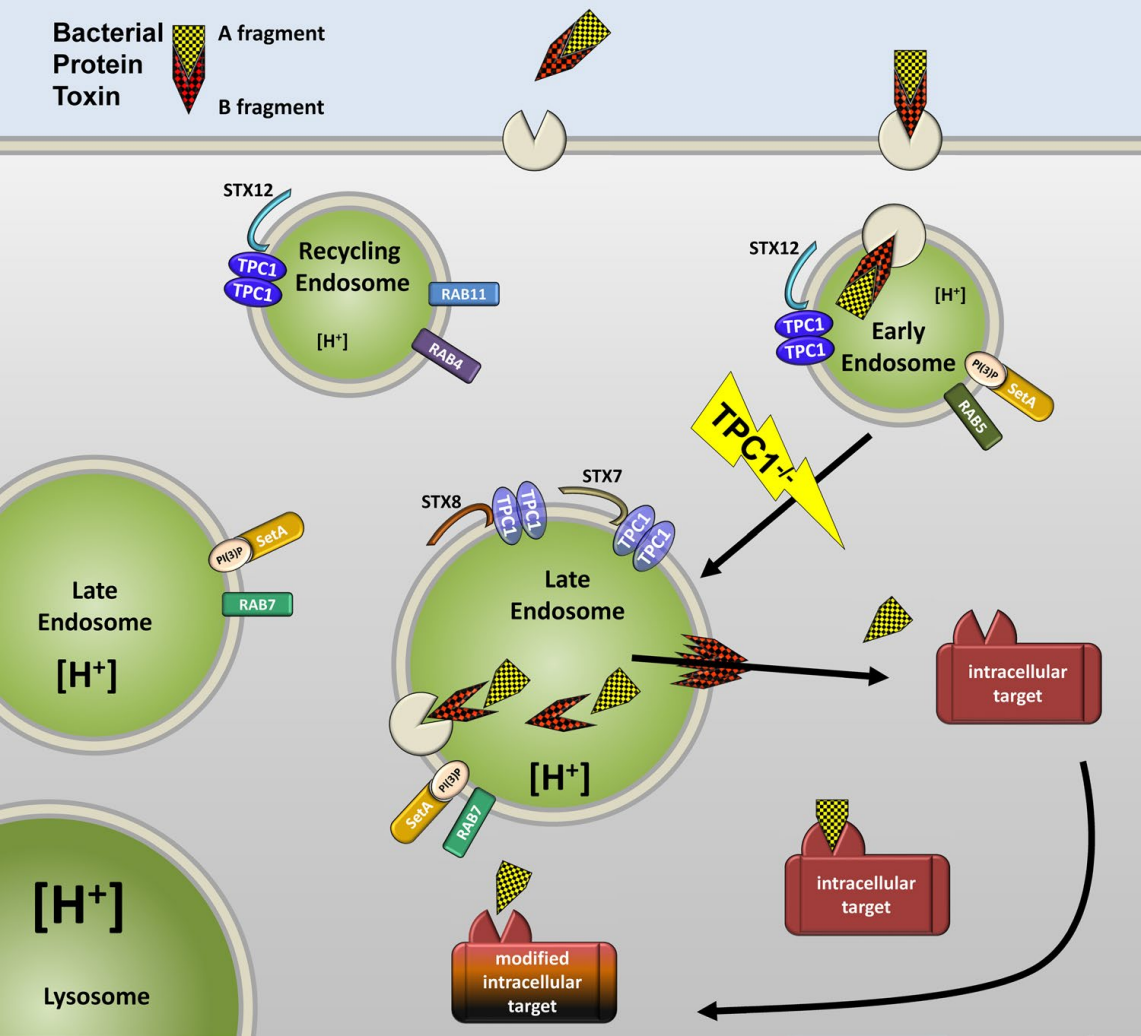
Cartoon illustrating the role of TPC1 for the uptake and translocation of short-trip toxins via early and late endosomes into the cytosol integrating the different experimental data (Figs 1–6). Distinguishable populations of endo-lysosomal compartments are drawn as vesicles of arbitrary size and shape with their characteristic markers and TPC1-interactors identified in this study. Accordingly, highest density of TPC1 is found in early and recycling endosomes, strongly overlapping with the distribution of syntaxin-12. Impaired fusion/transition from early to late endosomes upon ablation of TPC1 stalls the release of active bacterial protein toxins.

All these results were integrated into a cellular model of TPC1 function and localization in endo-lysosomal compartments (Fig. 7). Whereas the role of TPC1 in early and recycling endosomes appears to be well supported by our data, its representation and function in late endosomes is less clear. Transition from early to late endosomes is likely a multistep process, and both TPC1-positive and TPC1-free pools of late endosomes may exist with different destinations of their cargo. The dependence of Ebola virus entry on both functional TPC1 and TPC2 suggest TPC channels acting in series along the endo-lysosomal pathway. However, it remains unclear at which step TPC2 takes over the function of TPC1, and how this switch may be related to the fate decision of lysosomal degradation versus recycling or retrograde trafficking. Noteworthy, interaction of an overlapping subset of syntaxins (6, 7 and 12) with TPC2 have been found by a recombinant approach^7^.

In the context of the kidney, the strong overlap of TPC1 with syntaxin-12 specifically below the apical membrane of proximal tubule cells (Fig. 6) indicates that TPC1 might be involved in early endocytic fusion processes that occur nearly exclusively at the apical membrane. Due to the importance of this nephron segment for reabsorption of a bulk of solutes we hypothesize that TPC1 may be involved in the regulation of expression density of transporters, ion channels or other exchange proteins at the apical membrane.

## Materials and Methods

### Materials

[^3^H]-labeled adenine was obtained from Perkin Elmer. [^32^P]-NAD^+^ was purchased from Hartmann Analytic GmbH. ComplexioLyte 72 was from Logopharm GmbH, Germany. Cholera toxin (CT) and diphtheria toxin (DT) were obtained from Sigma; *Pasteurella multocida* toxin (PMT) was purified as described in ref. 52 and anthrax protective antigen and lethal factor as in ref. 53.

Phospho-ERK antibody (Phospho-p44/42 MAPK (Thr202/Tyr204)) was obtained from Cell Signaling (mAb #4370) and anti-GFP antibody from Abcam (#6658). *Lotus tetragonolobus* lectin (LTL) was purchased from Vector laboratories.

### Generation of TPC1 antibodies

TPC1 antibodies were generated against two different N-terminal Cys-peptides (peptides were obtained from Intavis) corresponding to C-terminal amino acid sequences from murine TPC1 peptide 1 (aa region 774–790; CYQEEIQEWYEEHAREQE-NH2) and peptide 2 (aa region 800–817; CGPAAQQPPGSRQRSQTVT-COOH) of murine TPC1 (NCBI RefSeq: NP_665852.1). Sera from immunized rabbits (peptide 1: batches #3526 and #3527, together referred to as anti-TPC1c, peptide 2: batches #832 (anti-TPC1a) and #836 (anti-TPC1b), Gramsch Laboratories) were peptide affinity purified employing the SulfoLink Immobilisation Kit for Peptides (Thermo Scientific). Purification was performed on an ÄKTAprime plus station (GE Healthcare Life Sciences) by packing the peptide coupled resin into Tricorn 10/100 columns (GE Healthcare Life Sciences) and eluting the antibodies by applying a linear pH gradient.

### Plasmids

The TPC1 open reading frame in TPC1-pcDNA3, TPC1-pEGFP-N1 and TPC1-ptd-Tomato-N1 was cloned from mouse brain cDNA. Stx7-pEGFP-N1, Stx8-pEGFP-N1 and Stx-12-pEGFP-N1 were all cloned from mouse kidney cDNA. A fluorescent PI(3,5)P2 probe was generated according to Li *et al*. by subcloning an ordered 435 bp gBlock (Integrated DNA Technologies) comprising an codon optimized sequence for the ML1N*2 tandem repeat into the pEGFP-C1 vector using BglII and BamHI (gBlock sequence: agatctacgatggccacacccgcgggacgaagagcttctgaaacggagagactgctcacacccaacccgggatacggaacccaggtgggaacttctccagcacctacgacgccgaccgaggaggaagacctgcggcgaagacttaaatacttcttcatgagtccttgcgataagttcagggccaagggacggaagccttgcaagctcatgctgcggatacttacaatggccacaccagctgggcggcgagcctctgagacagagagactcctgacacctaacccaggctacggaacacaggtgggaacatctccggcacccacaaccccgaccgaggaggaggatttgaggcggagactcaaatacttcttcatgtctccatgcgacaagttccgagccaaaggaagaaaaccctgtaagttgatgctggatcc). Rab6-GFP was kindly provided by Dr. Julia Blümer, Max Planck Institute of Molecular Physiology, Dortmund. Rab4-mCherry was obtained from the Toolbox Freiburg (Plasmid 880, principal investigator: Dr. Winfried Römer). EGFP-SetA^301–644^ ^31^ was kindly provided by Thomas Jank, Institute of Pharmacology and Toxicology, Freiburg. pEGFP-C1-SidC-P4C was cloned from the pGEX-4T-1-SidC^609–776^ plasmid^54^ which was kindly provided by Dr. Hubert Hilbi, Max von Pettenkofer Institut, München. The following plasmids were obtained from Addgene: Rab11-GFP^55^ (ID: 12674, principal investigator: Richard Pagano), Rab7-GFP^55^ (ID: 12605, principal investigator: Richard Pagano), DsRed-Rab5^56^ (ID: 13050, principal investigator: Richard Pagano), pEGFP-LC3^57^ (ID: 21073, principal investigator: Tamotsu Yoshimori) and hCas9^25^(ID: 41815, principal investigator: George Church)). pEGFP-C1-Rab5 was derived from the DsRed-Rab5 plasmid.

### Cas9 mediated TPC1 gene editing

The U6 target gRNA expression vector was assembled according to the protocol from ref. 25. Briefly, a target sequence within Exon 8 (GATGTCCATGAAGGGCGGCAGGG) of human TPCN1 and exon 5 (CGTCCGGCACAAACGTACCATGG) of murine TPCN1 was chosen. These unique guide DNA sequences were incorporated into the DNA building block as described by ref. 25. An entire 455 bp fragment including the human U6 polymerase III promoter and the final guide RNA sequence was ordered from Integrated DNA Technologies and cloned into the pCR-BluntII-TOPO vector (Invitrogen). The hCas9 plasmid was ordered from Addgene ID: 41815. Cells were co-transfected with both plasmids.

Following selection with G418 and repeated changes of media, single cell clones were recovered and separated. Primers flanking either hTPCN1 Exon 8 or mTPCN1 Exon 5 were used for PCR amplification and analysis of the modified regions. Verification of TPC1 gene editing was performed by sequencing and by Western blot analysis using an anti-TPC1 (#3526) antibody.

### Quantification of PMT uptake by MEF cells

MEF cells were grown on HCl-washed glass coverslips to a confluence of 60 to 80% and intoxicated with 0.5 µM Atto568 labeled PMT for 7.5, 15, 30 or 45 minutes. Cells were briefly washed with PBS and immediately fixed with 4% paraformaldehyde in PBS for 20 minutes at room temperature. After washing three times with PBS cells were mounted with FluoProtect + Hoechst (HYPERMOL, Bielefeld, Germany). Images were acquired with a Zeiss 200 M confocal microscope.

Image processing and quantification of PMT-Atto568 uptake was performed with ImageJ software. Background was subtracted using the implemented rolling ball algorithm. Borders of single cells were designated with the freehand selection tool. Cell sizes were calculated. For intracellular particle analysis binary images were created. Thresholds for binary images were determined by using the Kapur-Sahoo-Wong (Maximum Entropy) thresholding method^58^. PMT-Atto568 positive vesicles were counted utilizing the particle analysis tool. The number of PMT-Atto568 positive vesicles per µm² cell area was calculated for each analyzed cell.

### Housing of animals

All animal experiments were performed in compliance with the German animal protection law (TierSchG). Mice were housed and handled in accordance with good animal practice as defined by FELASA (www.felasa.eu) and the national animal welfare body GV-SOLAS (www.gv-solas.de). The animal welfare committee of the University of Freiburg as well as local authorities (Regierungspräsidium Freiburg) approved all animal experiments. Animals were housed in a temperature and humidity controlled vivarium with a 12 h light-dark cycle, food and water were available ad libitum.

### TPC1 tissue expression and deglycosylation assay

Mice were sacrificed by cervical dislocation. Tissue samples were cut into small pieces and homogenized in 90% (v/w) homogenization buffer HB (10 mM Tris, 250 mM Sucrose, 5 mM EDTA, pH 7.6, supplemented with Roche Complete protease inhibitor mix) using an Ultra-Torrax homogenizer (IKA T10 basic). Homogenates were centrifuged for 20 min at 2,800 × g. The pellet was resuspended in HB and centrifugation was repeated. The combined supernatants were mixed with an equal volume of carbonate extraction buffer CEB (100 mM Na_2_CO_3_, pH 11) and incubated for 1 h at 4 °C. Extracts were centrifuged for 1 h using a Beckmann SS-34 rotor at 20,000 rpm. Pellet was washed with 10 mM Tris, dissolved in Glycoprotein denaturation buffer (NEB) and heated for 5 min at 80 °C. Protein concentration was measured using the RC/DC Kit from Bio-Rad and adjusted to 1.5 µg/µl. A deglycosylation assay was performed using the PNGase-F deglycosylation kit from NEB. According to the manufacturer’s protocol 30 µg of each tissue sample were incubated for one hour at 37 °C in the presence or absence of 1000 U PNGase-F. Samples were mixed with 25% of 5x Loading buffer (45% gylcerol, 12% SDS, 200 mM DTT, 200 mM Tris-HCl, pH 6.8, 0,09% Bromophenol blue) and heated for 5 min at 80 °C prior to Western blot analysis.

### Immunohistochemistry

Deeply anesthetized mice were perfused through the left ventricle with 0.9% NaCl, followed by 4% paraformaldehyde in 0.1 M phosphate buffer, pH 7.4. Kidneys were removed and postfixed overnight in the same fixative before Cryostat sectioning (8 µm). For antigen retrieval kidney cryosections were treated with citrate buffer (pH 6, microwave heating for 10 min) and then incubated with blocking solution (phosphate buffer containing 1% Triton X-100, 1% bovine serum albumin and 5% normal goat serum) for 1–2 h. Sections were incubated for 3 h at room temperature with either the anti-TPC1 antibody (5 µg/ml in blocking solution) and anti-Syntaxin-12 (monoclonal mouse, Synaptic Systems GmbH; 2 µg/ml in blocking solution) or anti-TPC1 and biotinylated *Lotus tetragonolobus* lectin (LTL, 10 µg/ml, FITC-conjugated from Vector Laboratories) followed by a 4 °C overnight incubation. After washing with phosphate buffer, sections were incubated with the secondary Ab (Cy3-biotinylated goat anti-rabbit IgG, diluted 1:500, goat anti-mouse conjugated Alexa 488 diluted 1:250) with 1% BSA and 0.1% Triton X-100 for 2 h at room temperature. Sections were then washed and stained with 4′,6-Diamidino-2-phenylindole [DAPI]. After final washes in phosphate buffer slices were mounted with Immu-Mount (Thermo Scientific Shandon). Images were taken with a Zeiss Axiovert 200 M microscope equipped with a Photometrics Coolsnap 2 digital camera controlled by Metamorph (Molecular Devices). Images were either processed by deconvolution or directly acquired by a Yokogava CSU-X1 spinning disc confocal.

### Cell culture and transfection

HeLa, MEF and J774 cells were grown in DMEM (Biochrom) containing 10% fetal calf serum (FCS) and 1 mM sodium pyruvate (PAA Laboratories) in an atmosphere of 5% CO_2_ at 37 °C. MDCK cells were grown in MEM Eagle (PAN-Biotech) containing 10% fetal calf serum (FCS) and 1% NEA 100x (Biochrom) in an atmosphere of 5% CO_2_ at 37 °C. Cells were passaged every two or three days. Cells were transfected one day after seeding at a confluency of about 60 to 70% with the appropriate expression vectors using 1 µl LipofectAMINE 2000 (Invitrogen) per µg DNA. Cells were incubated for one or two days following transfection before immunocytochemical or biochemical experiments. All cell lines were regularly tested for *Mycoplasma* contamination.

### Co-localization studies and correlation analysis of TPC1 with subcellular markers

HeLa cells were washed once with PBS and fixated with 4% paraformaldehyde for 15 min at RT. Cells were then washed three times with PBS and directly embedded in ProLong Gold (Invitrogen) except for LAMP1 stained cells. Immuncytochemical staining was performed overnight at 4 °C with an anti-LAMP1 antibody (H4A3 Santa Cruz) at an concentration of 1 µg/µl in PBS after an initial permeabilization and blocking step (0.1% Triton and 4% NGS in PBS) for 30 min at RT. After three washing steps with PBS a 1:500 dilution of the secondary goat Alexa 568 anti-mouse antibody (Invitrogen) was applied for one hour at RT prior to the washing and embedding step.

Image acquisition was performed with a Zeiss 200 M confocal microscope equipped with a CSU-X1 Spinning Disc (Yokogawa). Image data processing and quantification were evaluated with two different methods: a co-localization and a Pearson correlation analysis. Each image consisted of two fluorescence channels: One for TPC1 and one for the intracellular endo-, lyso- or phagosomal marker or TPC1 as a reference. Co-localization analysis was performed by using the image software Metamorph 7.711. To obtain the co-localization value, fluorescence signals were converted from grayscale to black and white by setting the intensity threshold level. The yielded value gave the percentage of covered subcellular area. To exclude any bias, fluorescence channel threshold levels of the given subcellular marker were set manually in a first step. In a second step, the threshold for TPC1 was adjusted that the retrieved covered cellular area was the same as for the intracellular marker used in the particular image. Co-localization was then received from both thresholded fluorescence channels.

Pearson correlation measurement was performed by using the image software Autoquant × 2. Two-dimensional histograms, showing the given pixel intensity combinations from both fluorescence channels, were generated for each image. For noise reduction, 70% of all pixels in the low-intensity range were cut off proportionally in both channels within the two-dimensional histogram. The remaining 30% “nonzero” pixels were used to calculate the Pearson correlation coefficient.

Co-localization values and Pearson correlation coefficients from all subcellular markers were compared by ANOVA against the measured reference values (TPC1-EGFP with TPC1-Tomato) by using GraphPad Prism 4.

### Heterologous Co-immunoprecipitation

Co-immunoprecipitation was essentially performed as described previously^59^. Briefly, confluent transfected MDCK cells from two wells of a six well plate were washed with ice cold PBS and harvested by scraping using 1 ml PBS (with Roche Complete). After sonication with 5–7 pulses (Bandelin Sonopuls HD60) homogenates were centrifuged for 10 min at 3,000 × g. Supernatants were taken off and solubilised by incubation with 250 µl CL-72 (Logopharm) for 2 h. After a final centrifugation for 30 min at 16,100 × g supernatants were directly employed for immunoprecipitation. For this samples were split into two equal fractions. One half was mixed with 4 µg immobilized anti-TPC1 antibodies, the other half with 4 µg immobilized rabbit control IgG’s (Abcam). Antibodies were immobilized by covalent coupling onto Dynabeads M-270 Epoxy (Invitrogen) at a concentration of 8 µg/mg beads. Co-immunoprecipitation was performed for 2 h. The remaining supernatant was saved for Western blot analysis. The solid support was washed three times with 10% CL72 (diluted in PBS) for 5 min. Elution was done twice with 15 µl SDS buffer without reducing agents at 37 °C for 15 min. The combined eluates were mixed with 3.3 µl β-mercaptoethanol and heated for 5 min at 80 °C.

### Immunoblot Analysis

For immunoblotting, samples were subjected to SDS-PAGE and transferred onto polyvinylidene difluoride-membrane. Tubulin antibody was purchased from Sigma-Aldrich (Taufkirchen, Germany). Deamidation specific antibody anti-Gα_q_ Q209E (3G3) was used as described before^19^. Binding of the appropriate horseradish peroxidase-coupled secondary antibody was detected by using enhanced chemiluminescent detection reagent and the imaging system LAS-3000 (Fujifilm, Düsseldorf, Germany). Quantifications of immunoblots were done using MultiGauge V 3.0 (Fujifilm, Düsseldorf, Germany).

### cAMP Accumulation Assay

cAMP levels were measured utilizing the cAMP Glo assay (Promega) as described in the manufacturer’s instructions.

### ADP-ribosylation of EF-2

For post ADP-ribosylation, cells were incubated with DT (100 pM) for indicated times. Thereafter cell lysates were acquired with RIPA buffer and post ADP-ribosylation was performed as decribed before^60^. Briefly, cell lysate was incubated for 30 min at 37 °C with 4.6 kBq radioactive ^32^P-NAD^+^ and activated DT (300 nM) in 20 μl ADP-ribosylation buffer (1 mM EDTA, 50 mM NaCl, 50 mM Tris-HCl (pH 7.5), 1 mM DTT and 5 mM thymidine). Radiolabeled proteins were detected by SDS-PAGE and subsequent phospho-imaging (Molecular Dynamics). Quantification of radiolabeled EF-2 was performed using Image Quant (Molecular Dynamics).

### Isolation of endosome-enriched mouse kidney membranes

Mice were sacrificed by cervical dislocation. The removed kidneys were homogenized with 10 volumes homogenization buffer HB (10 mM Tris pH 7.6, 250 mM Sucrose, 5 mM EDTA, Complete) in a glas potter by applying ∼15 strokes. The crude homogenate was centrifuged for 10 min at 1000 × g. The received supernatant was transferred into an Ultra-Clear Centrifuge Tube (25 × 89 mm, Beckmann Coulter) and slowly underlayed with 12 ml of a 22.75% sucrose solution (10 mM Tris pH 7.6, 5 mM EDTA) and 6 ml of a 46% sucrose solution (10 mM Tris pH 7.6, 5 mM EDTA). After gradient centrifugation in a SW32Ti swing bucket rotor (Beckman Coulter) for 1.5 h at 31,000 rpm the obtained band on top of the 46% sucrose cussion was collected py punctuating the gradient tube with a Venofix A 19 G L:30 cm infusion needle (B. Braun). The collected membrane fraction was dialysed against TBS overnight and its protein concentration was assessed with the RC DC Proteinassay Kit (Biorad). Finally, aliquots were centrifuged at 16,100 × g for 1 h and the obtained pellets were snap-frozen for further processing.

### AP-MS analysis of native TPC1

For TPC1 affinity purification, membranes prepared from wildtype and TPC1 knockout mouse kidney were solubilized with ComplexioLyte 72 (Logopharm GmbH) detergent buffer (1 ml per mg membrane protein, supplemented with protease inhibitors and 1 mM EDTA). After removal of non-solubilized material by centrifugation at 16,100 × g for 30 min, the retrieved solubilisate was incubated for 2 h with immobilized antibodies (3.9 µg antibody per mg of solubilised membrane; Dynabeads antibody coupling kit from LifeTechnologies). The beads were washed twice with 0.1 × CL-72 diluted in PBS. Elution was done in SDS buffer. Aliquots were taken along this procedure for SDS-PAGE-Western blot analysis (Fig. 4b).

AP eluates were briefly run on SDS-PAGE gels, silver-stained and the gel lanes excised in two sections subjected to in-gel tryptic digestion (sequencing grade modified trypsin, Promega, Germany; 1:200 in 25 mM NH_4_HCO_3_) as described in ref. 61. Extracted peptides were vacuum dried, redissolved in 0.5% (v/v) trifluoroacetic acid and aspirated by the autosampler of a split-based UltiMate 3000 HPLC (Dionex/now Thermo Fisher Scientific). Peptides were trapped on a C18 PepMap100 pre-column (5-µm particles; Dionex/now Thermo Fisher Scientific)- with eluent A (0.5% (v/v) acetic acid for 5 min at 20 µl/min and separated via a 10-cm C18 capillary columns (PicoTip emitters (75 µm i.d. with 8-µm tip; New Objective) self-packed with ReproSil-Pur 120 ODS-3 (3 µm particles, Dr. Maisch HPLC); flow rate 300 nl/min) by continuously increasing the percentage of eluent B (0.5% acetic acid in 80% acetonitrile; A/B gradient: 5 min 3% B, 60 min from 3% B to 30% B, 15 min from 30% B to 100% B, 5 min 100% B, 5 min from 100% B to 3% B, 15 min 3% B). Eluting peptides were electrosprayed at 2.3 kV (positive ion mode) into an LTQ FT Ultra mass spectrometer (Thermo Scientific) with a nano-electrospray ion source (Proxeon Biosystems/now Thermo Scientific) using the following settings. Capillary temperature was kept at 125 °C, precursor spectra (scan range 370–1,700 m/z) used for quantification were acquired with a target value of 1,000,000 and a nominal resolution of 100,000 (FWHM). Up to 5 data-dependent CID fragment spectra (most intense ions) per scan cycle were acquired in the ion trap with a target value of 10,000 (maximum injection time 400 ms, isolation width 2.0 Th, normalized collision energy 35, activation Q 0.25, activation time 30 ms) with injection waveforms, dynamic exclusion (repeat count 1; exclusion list size 500; repeat/exclusion duration 30 s; exclusion mass width ± 20 ppm), preview mode for FTMS master scans, charge state screening, mono-isotopic precursor selection, and charge state + 1 rejection enabled.

LC-MS/MS data were extracted using “extract_msn.exe” (grouping tolerance 0.05; Thermo Fisher Scientific). Peak lists were first searched against UniProtKB/Swiss-Prot (release 2012_01; all canonical mouse, rat and human entries as well as P00761|TRYP_PIG, P00766|CTRA_BOVIN and P02769|ALBU_BOVIN) with peptide mass tolerance ± 15 ppm using Mascot 2.3 (Matrix Science). After linear shift mass recalibration by a home-made script, precursor tolerances were reduced to ± 5 ppm for final searches against an equivalent (more recent) database (UniProt release 2016_07; Mascot 2.5.1). Final searches used the following settings: fragment mass tolerance ± 0.8 Da, one missed trypsin cleavage and common variable modifications (Acetyl (Protein N-term), Carbamidomethyl (C), Gln- > pyro-Glu (N-term Q), Glu- > pyro-Glu (N-term E), Oxidation (M)), Phospho (S/T/Y) and Propionamide (C) accepted, and significance threshold P < 0.05). Proteins identified by only one specific MS/MS spectrum or representing exogenous contaminations such as keratins or immunoglobulins were eliminated.

Relative coverage of protein amino acid sequences by MS-identified peptides (Supplemental Figure 4c) was calculated as SC = N_i_/(N_i_ + N_an_), where N_i_ is the number of amino acid residues covered by identified peptides (max. expect value 0.05) and N_an_ is the number of MS-accessible (i.e. tryptic peptides within 738 < MW < 3000) but not identified amino acids in the given sequence.

Quantitative evaluation of AP-MS data was based on Peak Volumes (PVs = peptide m/z signal intensities integrated over elution time) following the procedure described in (Bildl *et al*. 2012). Briefly, PVs were calculated from LC-MS (FT) full scans using MaxQuant (v1.4.1.2, http://www.maxquant.org) with integrated offline mass calibration, aligned between different LC-MS/MS runs and assigned to the peptides identified by Mascot (retention time tolerance ± 1 min, mass tolerance: ± 2.5 ppm) using an in-house developed software. Relative protein abundance in target AP versus target knockout control (rPV) was determined using the TopCorr method (median of at least 2–6 individual peptide PV ratios of the best correlating protein-specific peptides)^62^. AP specificity thresholds were then determined from rPV histograms of all proteins detected in the respective AP versus control. Proteins were considered specifically (and consistently) co-purified when rPV(wild-type mouse versus IgG and versus knockout)/threshold(versus IgG and knockout) were >1 with both antibodies used. For visualization of AP consistency and enrichment of TPC1, protein abundances were estimated by their abundance_norm(spec)_ values, calculated as the sum of all protein-specific peptide PVs divided by the number of amino acids from the respective protein sequence accessible to MS analysis under the conditions used (Supplemental Figure S4c, ref. 62).

## Electronic supplementary material

Supplementary figures and table
