# Supplementary material for: The two-pore channel TPC1 is required for efficient protein processing through early and recycling endosomes
Source: Sci Rep. 2017 Aug 30;7:10038. doi: 10.1038/s41598-017-10607-4 (PMC5577145; doi:10.1038/s41598-017-10607-4)
Supplement: Supplementary file 1 — Supplementary figures and table [file 41598_2017_10607_MOESM1_ESM.pdf]

The two-pore channel TPC1 is required for efficient protein processing through early and recycling endosomes

Jan Castonguay[1], Joachim H. C. Orth[1], Thomas Müller[1], Faten Sleman[1], Christian Grimm[5], Christian Wahl-Schott[5], Martin Biel[5], Robert Theodor Mallmann[1], Wolfgang Bildl[2], Uwe Schulte[2,3,4], Norbert Klugbauer[1*]

[1]Institute of Experimental and Clinical Pharmacology and Toxicology, Faculty of Medicine, Albert-Ludwigs-University, Albertstrasse 25, 79104 Freiburg, Germany

[2]Institute of Physiology II, Faculty of Medicine, Albert-Ludwigs-University, Hermann-Herder-Strasse 7, 79104 Freiburg, Germany

[3]Logopharm GmbH, Schlossstrasse 14, 79232 March-Buchheim, Germany,

[4]Center for Biological Signaling Studies (BIOSS), Schänzlestrasse 18, 79104 Freiburg, Germany

[5]Department of Pharmacy, Center for Drug Research and Center for Integrated Protein Science Munich (CIPSM), Ludwig-Maximilians-University, Munich, Germany

Online supplemental material

Supplemental Figure S1

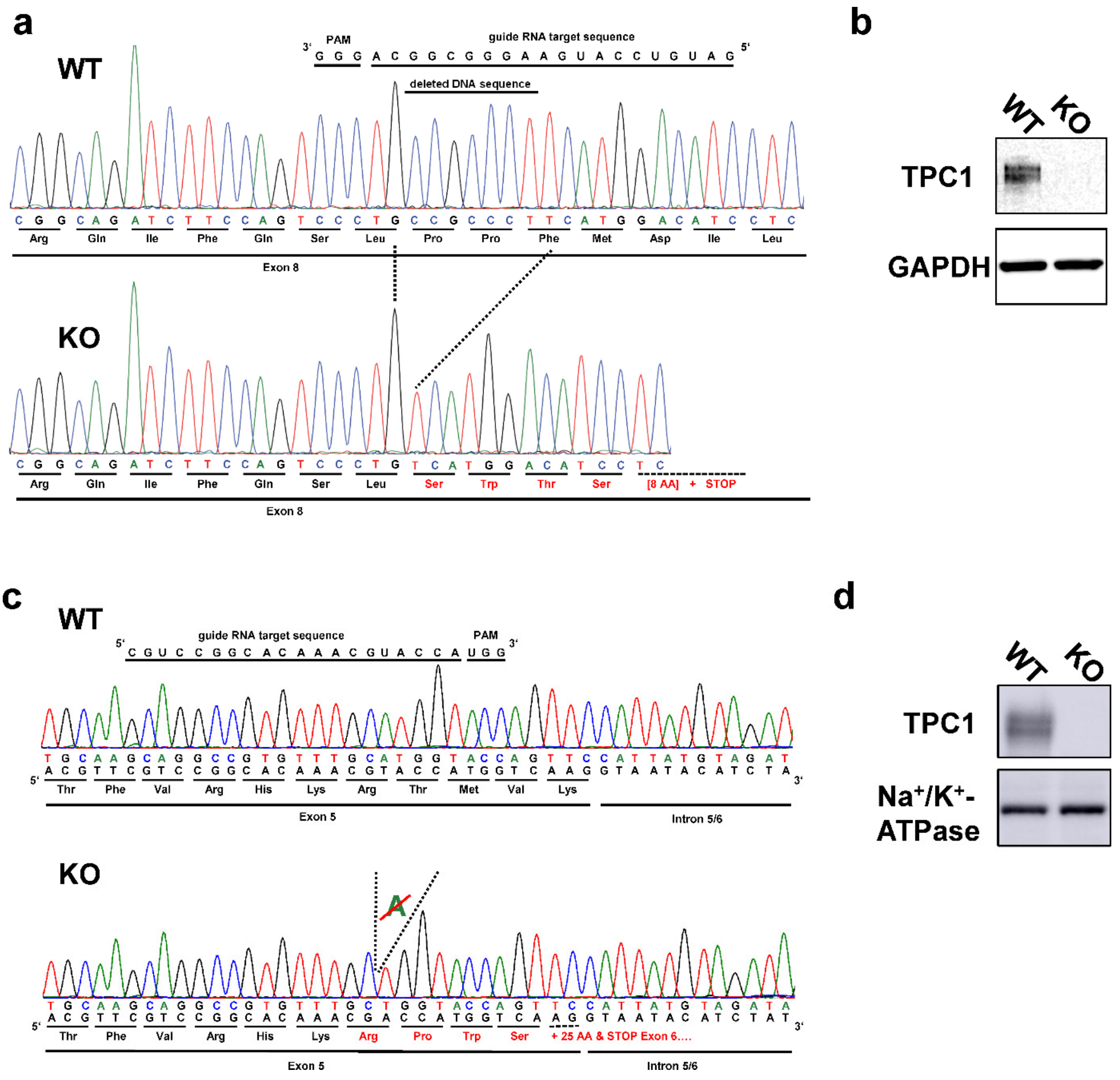

CRISPR-Cas mediated TPC1 gene editing in HeLa and J774 cells. (a) Western blot analysis of wild type (WT) and targeted HeLa (KO) cells using the TPC1 antibody. GAPDH was used as loading control. (b) Nucleotide and corresponding peptide sequence of part of exon 8 of the TPC1 gene that was targeted by the guide RNA indicated. WT shows the sequences of a non-targeted HeLa cell, KO that of a targeted cell clone. CRISPR-Cas mediated editing caused a 7 bp deletion and a frame-shift in the TPC1 cDNA that results in a premature stop codon. (c) Same as (a), but for J774 cells. (d) Nucleotide and

corresponding peptide sequence of part of exon 6 of the TPC1 gene that was targeted by the guide RNA indicated. WT shows the sequences of a non-targeted J774 cell, KO that of a targeted cell clone. CRISPR-Cas mediated editing caused a deletion of an A within exon 6 and thereby introduced a frame-shift in the TPC1 cDNA resulting in a premature stop codon.

Supplemental Figure S2

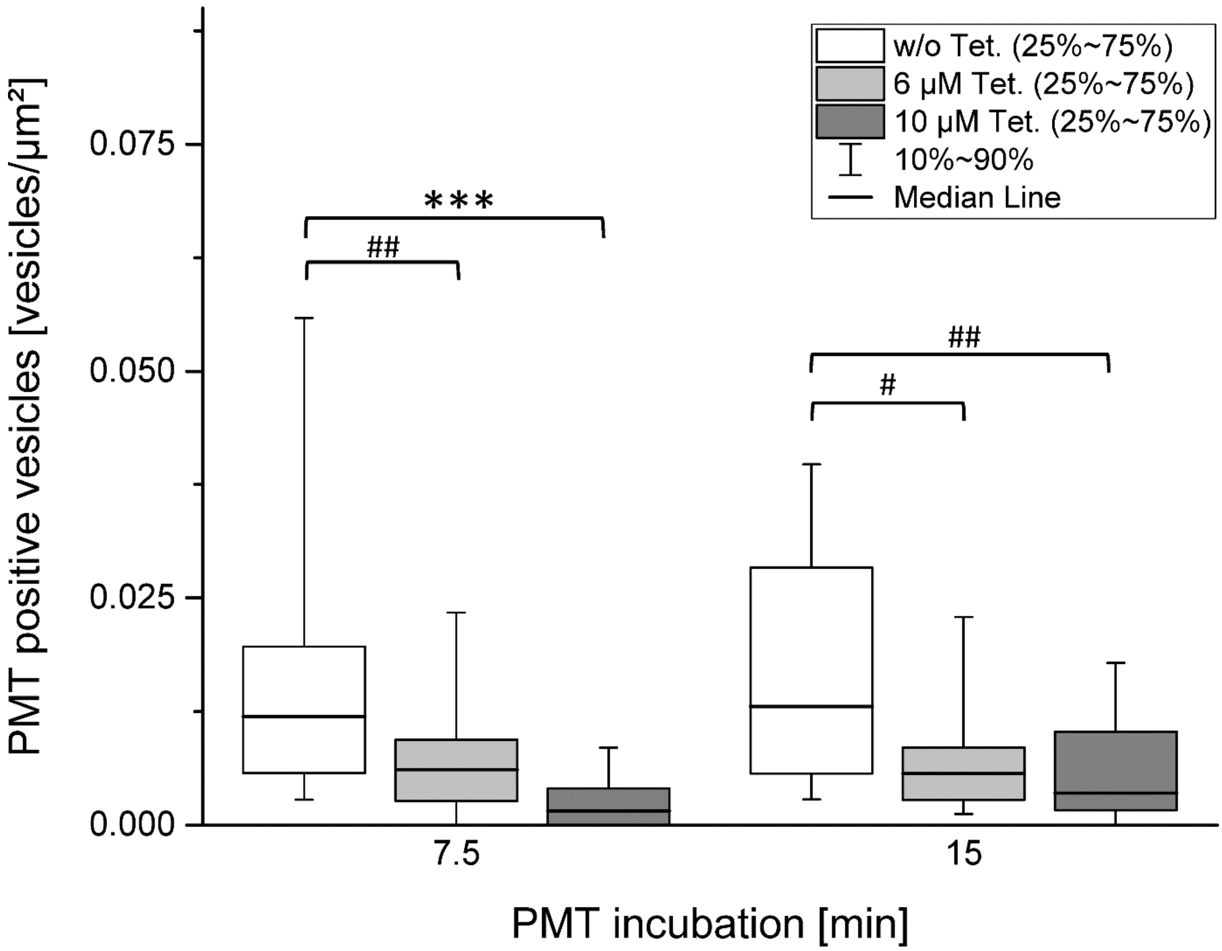

Uptake of PMT in the presence of 6 µM and 10 µM tetrandrine. Quantification of toxin uptake was performed by counting PMT positive vesicles per cell size (described in Materials and Methods). *** P<0,001; ## P<0,03; # P<0,05

Supplemental Figure S3

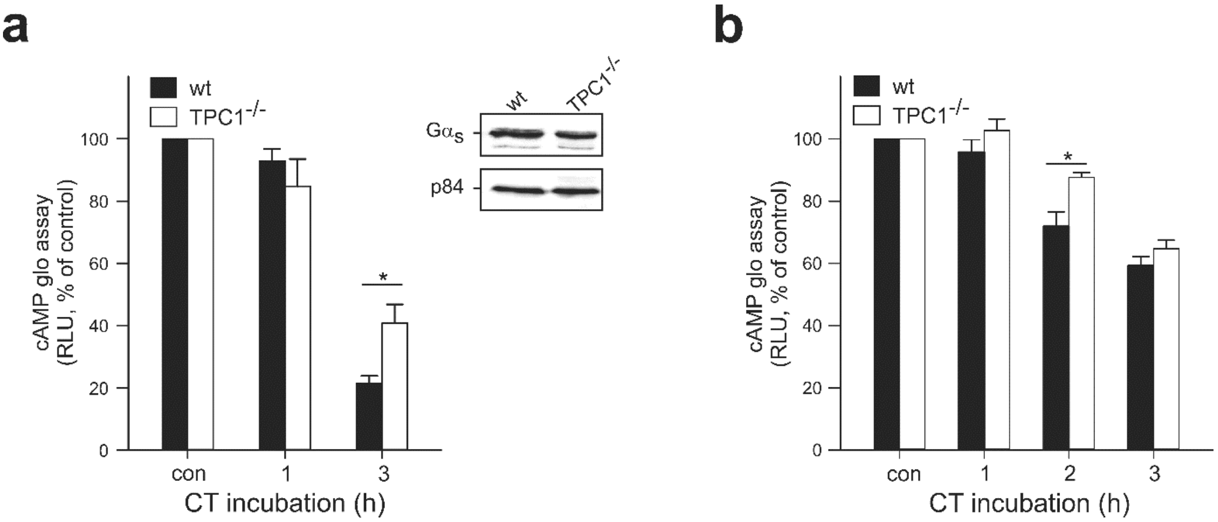

The biochemical activity of CT is not dependent on TPC1. (a, left) WT-MEF or MEF deficient for TPC1 (TPC1-/-) were incubated with CT (1 ng/ml) for indicated times. cAMP levels were determined using the cAMP Glo assay (Promega). This assay measures ATP-dependent luciferase activity leading to an inverse correlation of relative light units (RLU) and cAMP levels (n ≥ 3, mean ± SEM). (a, right) Western blot analysis of Gαs in WT-MEF or MEF deficient for TPC1. p84 was used as loading control to show the presence and equal content of $G\alpha_s$ in the MEFs tested. (b) CT-induced cAMP measurement in WT-HeLa cells and TPC1-knock-out HeLa cells (TPC1-/-). Cells were incubated with CT (300 ng/ml) for indicated times and cAMP levels were determined using the cAMP Glo assay as in (a) (n ≥ 3, mean ± SEM).

Supplemental Figure S4

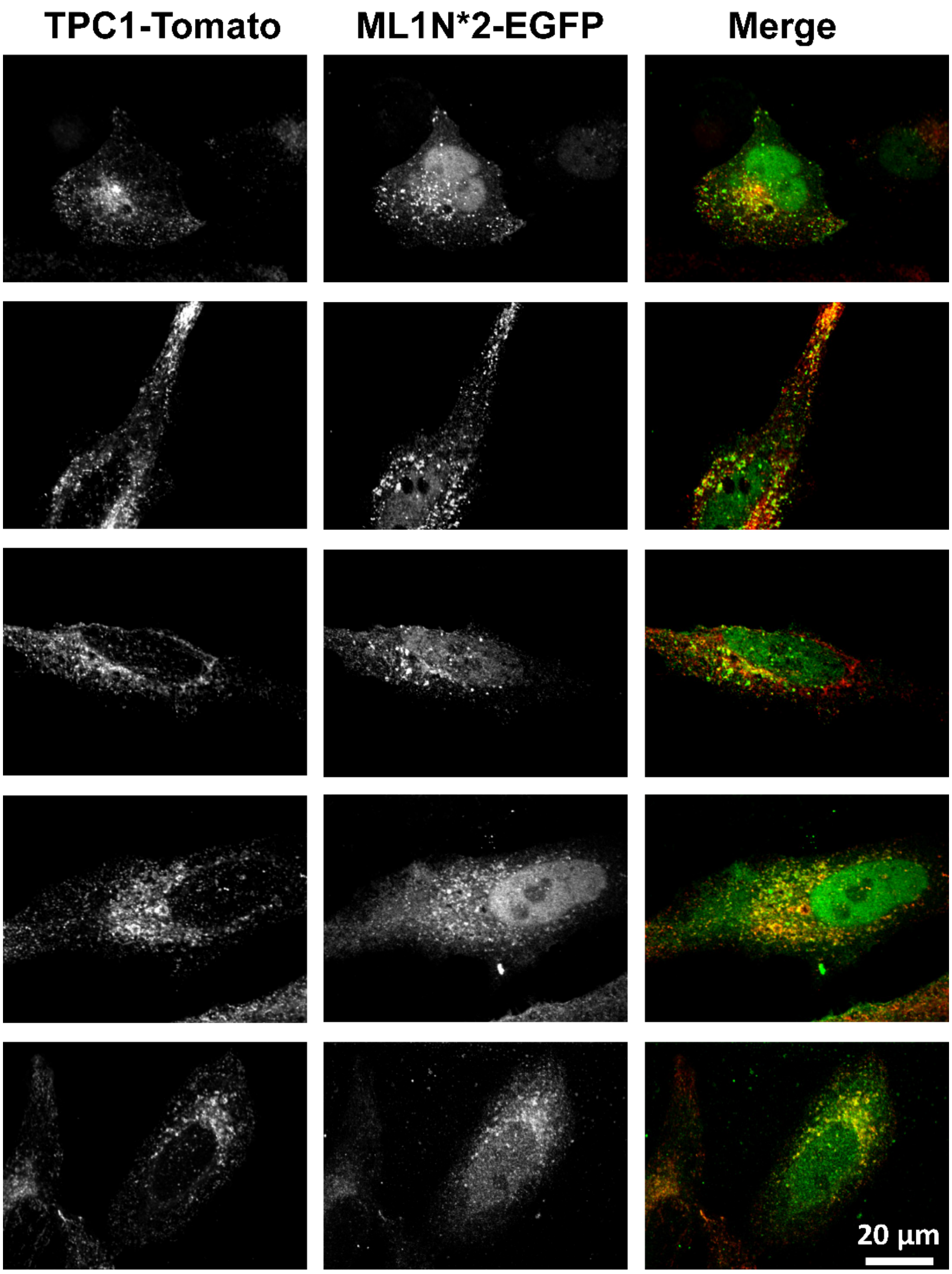

Co-localization analysis of TPC1-Tomato with ML1N*2-EGFP. The examples reflect the non-uniform distribution of the PtdIns(3,5)P_2 probe ranging from cells with a broad to a more punctate PtdIns(3,5)P_2 probe staining. Cells with a broad and more ramified pattern of the PtdIns(3,5)P_2 probe showed a high degree of co-localization, whereas cells that showed a more punctate PtdIns(3,5)P_2 probe staining demonstrated a low co-localization with TPC1.

a mouse kidney membrane prefractionation

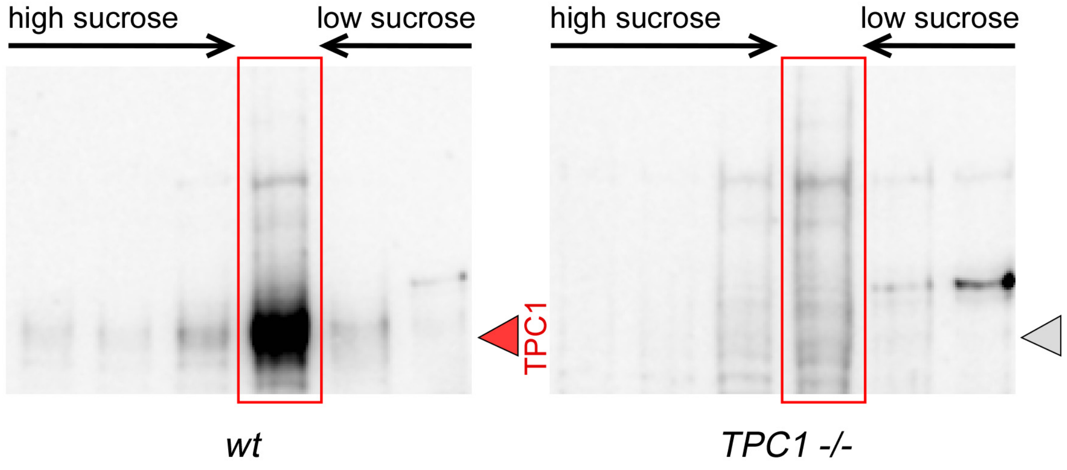

b

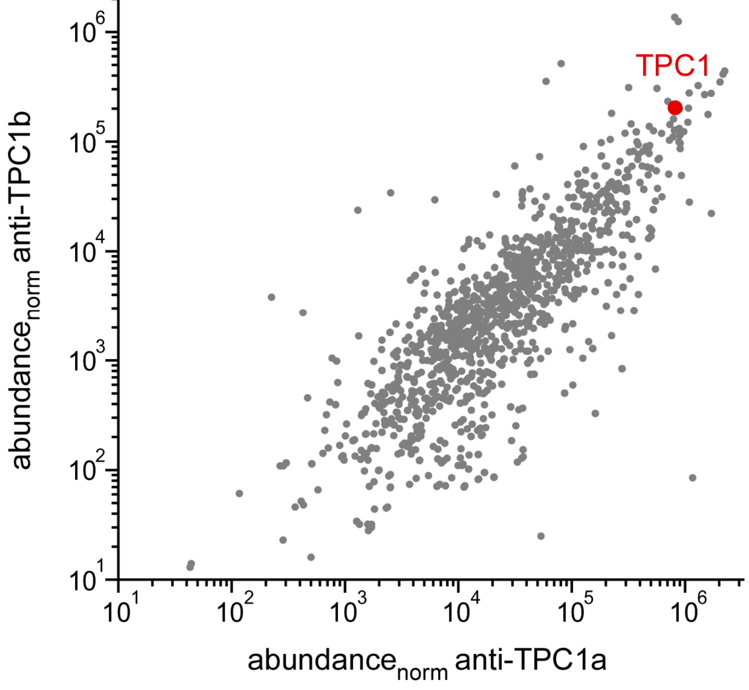

c

TPC1_MOUSE (Q9EQJ0) - Two Pore Calcium Channel Protein 1
Coverage 22.8% absolute, 54.2% relative, 817 aa

```
001 MAVSLDDDVP LILTLDEAES APLPPSNSLG QEQLPSKNGG SHSIHNSQVP
051 SLVSGADSPP SSPTGHNWEM NYQEAAIYLQ EGQNNDKFFT HPKDARALAA
101 YLFVHNHFFY MMELLTALLL LLLSLCESPA VPVLKLHTYV HATLELFALM
151 VVVFELCMKL RWLGFHTFVR HKRTMVKTSV LVVQFIEAIV VLVRQTSHVR
201 VTRALRCIFL VDCRYCGGVR RNLRQIFQSL PPFMDILLLL LFFMIIFAIL
251 GFYLFSTNPS DPYFSTLENS IVNLFVLLTT ANFPDVMMPS YSRNPWSCVF
301 FIVYLSIELY FIMNLLLAVV FDTFNDIEKH KFKSLLLHKR TAIQHAYGLL
351 ASQRRPAGIS YRQFEGLMRF YKPRMSARER FLTFKALNQS NTPLLSLKDF
401 YDIYEVAALQ WKAKRNRQHW FDELPRTAFL IFKGINILVN SKAFQYFMYL
451 VVAVNGVWIL VETFMLKGGN FTSKHVPWSY LVFLTIYGVE LFMKVAGLGP
501 VEYLSSGWNL FDFSVTAFAF LGLLALTLNM EPFYFIVVLR PLQLLRLFKL
551 KKRYRNVLDT MFELLPRMAS LGLTLLTFYY SFAIVGMEFF NGRLTPNCCN
601 TSTVADAYRF INHTVGNKTK VEEGYYYLNN FDNILNSFVT LFELTVVNNW
651 YIIMEGVTSQ TSHWSRLYFM TFYIVTMVVM TIIVAFILEA FVFRMNYSRK
701 SQDSEVDSGI VIEKEMSKEE LMAVLELYRE ERGTSSDVTR LLDTLSQMEK
751 YQQNSMVFLG RRSRTKSDLS LKMYQEEIQE WYEEHAREQE QQKLRGSVPG
801 PAAQQPPGSR QRSQTVT
```

Supplemental Figure S5

AP-MS analysis of TPC1 channels from mouse kidney. (a) Western blot analysis of sucrose gradient fractions resolving membrane vesicles from mouse WT (left) and TPC1 KO (right) kidneys probed with anti-TPC1 (#3526). Note that the total TPC1-specific immunoreactivity was retained in a single gradient fraction (boxed red) that was used for subsequent APs. (b) 2D plot of protein abundances determined by LC-MS analysis ($abundance_{norm}$ values, see Methods) in anti-TPC1 APs from mouse kidney with the indicated antibodies. Distribution of the proteins (depicted as grey dots) along a diagonal axis with m = 1 demonstrates the overall high consistency of the two AP datasets but also shows a significant amount of background proteins. (c) Absolute and relative coverage of TPC1 primary sequence by MS/MS-identified peptides (highlighted in red); sequence stretches not identified but accessible or inaccessible to MS analysis are depicted in black and grey, respectively.

Supplemental Table S1

Table S1 Proteins specifically and consistently co-purified with TPC1 in mouse kidney

Protein ID	Protein Name	Acc. No. (Uniprot)	MS/MS spectra αTPC1a/bwt	MS/MS spectra KOa/b	Classification
TPC1	Two pore calcium channel protein 1	Q9EQJ0	46/53	0/0	TPC pore-forming
STX7	Syntaxin-7	O70439	3/2	0/0	SNARE complex
STX8	Syntaxin-8	O88983	5/3	2/0	SNARE complex
STX12	Syntaxin-12	Q9ER00	4/4	0/0	SNARE complex
VTI1B	Vesicle transport through interaction with t-SNAREs homolog 1B	O88384	6/6	1/0	SNARE complex
IST1	IST1 homolog	Q9CX00	2/4	0/0	ESCRT machinery
CHM2B	Charged multivesicular body protein 2b	Q8BJF9	2/5	1/0	ESCRT machinery
CHMP3	Charged multivesicular body protein 3	Q9CQ10	2/4	1/0	ESCRT machinery
EAA3	Excitatory amino acid transporter 3	P51906	1/4	0/0	transporter
VATC1	V-type proton ATPase subunit C 1	Q9Z1G3	6/(3)	0/0	transporter
VATL	V-type proton ATPase 16 kDa proteolipid subunit	P63082	3/2	0/0	transporter
AQP1	Aquaporin-1	Q02013	3/6	2/2	channel
LAMP2	Lysosome-associated membrane glycoprotein 2	P17047	3/2	0/0	other
NUDT7	Peroxisomal coenzyme A diphosphatase NUDT7	Q99P30	1/5	1/1	other
EIF3H	Eukaryotic translation initiation factor 3 subunit H	Q91WK2	6/2	1/0	other
SERC1	Serine incorporator 1	Q9QZI8	18/14	0/0	uncharacterized
K1468	LisH domain and HEAT repeat-containing protein KIAA1468	Q148V7	(2)/4	0/0	uncharacterized

Proteins consistently and specifically co-purified with anti-TPC1 a and b (related to Fig. 4C, see also Methods). Columns (from left to right) indicate UniProtKB/Swiss-Prot identifier, protein name, database accession, number of MS/MS-assigned spectra in the anti-TPC1a or TPC1b APs from mouse wildtype and TPC1 knockout kidney membranes, and functional classification based on UniProtKB/Swiss-Prot. Grey numbers in brackets denote the number of assigned LC-MS features when no MS/MS spectrum was identified for the respective protein/AP dataset.
